# Anxiolysis for laceration repair in children: study protocol for an open-label multicenter adaptive trial (ALICE)

**DOI:** 10.1371/journal.pone.0324515

**Published:** 2025-06-04

**Authors:** Naveen Poonai, Vinolia Arthur-Hayward, Samina Ali, Vikram Sabhaney, Quynh Doan, Evelyne Trottier, Jocelyn Gravel, Nam Anh Tran, Maala Bhatt, Mohamed Eltorki, Jennifer Thull-Freedman, Julie Leung, Darcy Beer, Arlene Jiang, Raju Poolacherla, Anna Heath

**Affiliations:** 1 Department of Pediatrics, Schulich School of Medicine and Dentistry, London, Ontario, Canada; 2 Department of Epidemiology & Biostatistics, Schulich School of Medicine and Dentistry, London, Ontario, Canada; 3 Children’s Health Research Institute, London Health Sciences Centre, London, Canada; 4 Department of Pediatrics, Faculty of Medicine & Dentistry, University of Alberta, Edmonton,; 5 Women and Children’s Health Research Institute, Faculty of Medicine & Dentistry, University of Alberta, Edmonton, Canada; 6 Department of Pediatrics, University of British Columbia, Vancouver, British Columbia, Canada; 7 British Columbia Children’s Hospital Research Institute, University of British Columbia, Vancouver, British Columbia, Canada; 8 Department of Paediatric Emergency Medicine, CHU Sainte-Justine, Université de Montréal, Montreal, Quebec, Canada; 9 Department of Biostatistics, McGill University, Montreal, Quebec, Canada; 10 Department of Pediatrics, University of Ottawa, Ottawa, Ontario, Canada; 11 Departments of Pediatrics and Emergency Medicine, University of Calgary, Calgary, Alberta, Canada; 12 Department of Pediatrics, University of Manitoba, Winnipeg, Manitoba, Canada; 13 Child Health Evaluative Sciences, Peter Gilgan Centre for Research and Learning, The Hospital for Sick Children, Toronto, Ontario, Canada; 14 Department of Anesthesia, Western University, London, Ontario, Canada; 15 Division of Biostatistics, Dalla Lana School of Public Health, University of Toronto, Toronto, Ontario, Canada; 16 Department of Statistical Science, University College London, London, United Kingdom; PLOS: Public Library of Science, UNITED KINGDOM OF GREAT BRITAIN AND NORTHERN IRELAND

## Abstract

**Background:**

Lacerations are the most common traumatic reason for children to visit an emergency department (ED), accounting for almost half of all procedures performed. Children experience considerable distress during laceration repair, despite routine application of local anesthetic. Pharmacologic anxiolysis may mitigate the negative practice of forcefully restraining a child, however, evidence for the most effective agent is lacking. We aim to determine the most effective anxiolytic agent for laceration repair in children.

**Methods:**

This is a multicentre, phase III, three-arm, adaptive, randomized, open-label, trial. We will include children 2–12 years with a single laceration requiring suture repair in the ED. Participants will be randomized to receive intranasal dexmedetomidine (IND) 3 mcg/kg, intranasal midazolam (INM) 0.4 mg/kg, or inhaled 50% nitrous oxide (N_2_O). The primary outcome is the weighted mean anxiolysis score using the Observational Scale of Behavioral Distress – Revised (OSBD-R) from initial positioning to tying of the last suture. Secondary outcomes include need for additional anxiolytic, need for physical restraint, adverse events (AEs), and delayed maladaptive behaviors. The primary analysis will be conducted by intention-to-treat. Results will report posterior means, standard deviations (SDs), and 95% high density posterior credible intervals for *Total Distress Score* on the OSBD-R. We will rank interventions based on the probability that an intervention is superior (P_*best*_) and the Surface Area Under the Cumulative Ranking Curve (SUCRA) to indicate relative anxiolytic efficacy. The mean difference in *Total Distress Score* and secondary outcomes will be estimated using Bayesian models.

**Ethics and dissemination:**

Ethics approval will be obtained from institutional review boards of the participating sites. Informed consent will be obtained from guardians of all participants in addition to assent from all participants. Study data will be submitted for publication.

**Trial registration:**

Clinicaltrials.gov NCT05383495

## Introduction

Laceration repairs account for almost 50% of emergency department (ED) procedures [1] in children, and are consistently distressing. Yet, the optimal anxiolytic agents remain unknown. Even with topical anesthetics [2,3], there is compelling evidence that most children experience moderate to severe distress during laceration repair [4–8], manifested as fear, crying, and physical resistance [6]. Certified child life specialists (CCLSs) can help manage distress [4] with distraction but are available in only half of Canadian paediatric EDs [9] and almost no general EDs. Further, most lacerations occur in children <10 years [7,10]. Up to 75% are on the face [7,10], making distraction difficult and the need for a fully cooperative patient is essential for good cosmesis. Procedural distress is associated with nightmares, tantrums, separation anxiety, aggression, and withdrawal in >50% of children for up to a week [11]. For ED procedures including laceration repair, children in 79% of EDs required physical restraint, mainly by nurses [12], despite the anxiolytic midazolam being available in >80% of cases [12]. Restraint has moral, ethical, legal, and safety implications for healthcare providers (HCPs) [13] and can result in protracted psychological stress [14] and physical trauma [15] for children and families. Qualitative data demonstrate that children can find restraint more distressing than procedural pain [16]. Poor needle-related experiences also lead to needle fears in adulthood [17], contributing to vaccine avoidance [18,19]. Procedural distress causing poor patient cooperation also leads to low job satisfaction among nurses [20].

While no universal standard of care exists, intranasal midazolam (INM) is the most commonly used anxiolytic for laceration repair in children [3] due to its safety profile [21] and low cost. However, its efficacy is uncertain [22] and up to 40% of children experience discomfort with administration [22]. While studies have shown INM to be superior to oral midazolam [23] and placebo [24], with adequate anxiolysis in 74% of participants [25], up to 86% of children still required physical restraint, even at high doses [24,26].

A 2020 systematic review found the sedative, intranasal dexmedetomidine (IND), had minor adverse events (AEs), was tolerated in >90% of children [27], and was superior to INM for initial positioning for laceration repair [28]. Administration of IND is also better tolerated than INM in children [29]. A 2023 systematic review also found nitrous oxide (N_2_O) was associated with minor AEs and was well tolerated [30]. N_2_O is also familiar to clinicians [31], and while its efficacy versus INM and IND is unknown, its rapid onset and offset (3–5 minutes) [32–34] would expedite patient flow in busy EDs. Given the importance of decreasing children’s distress during laceration repair and the inconclusive evidence for INM, our primary objective is to determine which among INM, IND, and N_2_O is associated with the least behavioral distress, using the Observational Scale of Behavioral Distress – Revised (OSBD-R), among children undergoing laceration repair in the ED. Our secondary objective is to determine the most cost-effective anxiolytic for laceration repair in children.

## Materials and methods

### Design

This is a four-centre, adaptive, randomized, open-label, comparative effectiveness trial (clinicaltrials.gov NCT05383495). We will follow the CONSORT [35] (clinical trials) and ACE extension [36] (adaptive trials) guidelines for conduct and reporting. The adaptation involves an interim futility analysis where 1 or 2 arms with subthreshold efficacy will be dropped to reduce the risk of a child being randomized to an inferior agent. This approach avoids separate trials of IND and N_2_O and instead ranks the three anxiolytics based on efficacy so healthcare providers (HCPs) can weigh the relative benefits with the resources available in their practice setting. This study protocol is reported using the SPIRIT 2013 Checklist [37] (S1 File; S2 File; S3 File). At the lead site, the trial protocol was approved by Western University’s Health Sciences Research Ethics Board on February 10, 2024 (approval number 2025-120985-104864). Ethics approvals from all sites can be found in Supplements S6-S8.

### Recruitment

Enrollment began at Children’s Hospital London Health Sciences Centre on Dec 19, 2023, will begin at BC Children’s Hospital and Stollery Children’s Hospital on April 7 and April 17, 2025, respectively. Enrollment is anticipated to begin at CHU Ste Justine on May 5, 2025. Potential participants will be screened and enrolled consecutively during the hours of research personnel availability (up to 8 hours per day, 7 days a week). Research personnel will seek obtain consent for screening and data collection. For patients who pass initial screening, research personnel will confirm eligibility with the ED physician or their designate (any clinician who has the capacity to assess, manage, and discharge a patient). If eligibility is confirmed, research personnel will explain the study protocol and seek informed consent (and assent when appropriate) (S4 File). Mature minor consent forms will be available for both accompanied and unaccompanied minors. Research personnel will record basic demographic features and eligibility criteria of all children 2–12 years with a laceration during their availability, whether randomized or not, to assess for enrolment bias.

We estimate recruiting 15 participants/month for 24 months based on each site’s ED census and number of eligible patients, lead site enrollment, and research personnel availability in a previous IND dose-finding trial [38]. Root cause analyses and education will be carried out if less than 50% of eligible children are enrolled over two months at any site. A one-month probation will be considered, with further education if enrolment remains at less than 50% for another two months. If enrollment remains less than 50%, we will consider replacing the underperforming site with another site.

### Setting

This study will be conducted in four pediatric EDs across Canada: i) Children’s Hospital at London Health Sciences Centre (London, Ontario) (lead site); ii) Stollery Children’s Hospital (Edmonton, Alberta); iii) BC Children’s Hospital (Vancouver, British Columbia); iv) CHU Ste Justine (Montreal, Quebec). The annual ED census for participating sites ranges from 40,000–70,000 patient visits.

### Eligibility

Children will be eligible if they meet all the following criteria:

i) Age 2–12.99 years. This age has the highest prevalence of lacerations in children [7,10] and the OSBD-R [39] is validated in children at least two years of age;
ii) Single or multiple lacerations (no more that 2 cm apart) for which the physician believes repair does not require intravenous sedation. Repairs requiring intravenous sedation are uncommon [7,10] and procedural sedatives may compound effects of study interventions;
iii) No concomitant fracture or dislocation that requires orthopedic manipulation;
iv) Repair to involve sutures and be performed by ED physician or their designate (trainee, nurse practitioner, etc.);
iv) Child or caregiver desires anxiolysis. This incorporates family preferences based on their prior experiences or beliefs about the need for anxiolysis;
v) Predicted to resist positioning for repair by the parent, child life specialist, bedside nurse, or physician.
vi) Local anesthesia with topical anesthetic such as lidocaine-epinephrine-tetracaine (LET) and/or infiltrated lidocaine and/or ring/thenar blocks as these are standard anesthetic options for ED laceration repair [5,9,40].

Exclusion criteria are as follows:

i) Hypersensitivity to any intervention
ii) Received an opioid, sedative, or anxiolytic within an hour prior to interventions
iii) Occlusion of at least one nostril due to polyps or septal deviation
iv) Hemodynamic abnormalities: bradycardia or hypotension within 2 standard deviations of age-related normal value
v) Contraindications to intranasal dexmedetomidine (IND): renal insufficiency, uncorrected cyanotic heart disease or mineralocorticoid deficiency, cardiac conduction disorder, pulmonary hypertension or edema, vitamin B12 or folate disorder, phenylketonuria, or psychosis
vi) Contraindications to sedation: respiratory instability or impaired level of consciousness
vii) Contraindications to nitrous oxide: pneumothorax or pneumoperitoneum
viii) Suspected or known pregnancy (as reported by the patient)
ix) Inability to use the Observational Scale of Behavioral Distress – Revised (OSBD-R) due to a motor deficit
x) Unable to comprehend study tasks in English or French in the absence of a native language interpreter
xi) Weight at least 65 kg to avoid under-dosing of intranasal midazolam (INM) or IND
x) American Society of Anesthesiologists class greater than II as these are contraindications to sedation in the emergency department
xii) Concomitant fracture or dislocation requiring orthopedic manipulation as these may require intravenous procedural sedation

### Interventions

Eligible participants will be randomized to INM, IND, or N_2_O with a 1:1:1 allocation ratio in the following doses: i) INM 0.4 mg/kg (Sandoz or Pfizer) to a maximum of 10 mg/2 mL administered as a single dose 15 minutes prior to laceration repair consistent with its onset of anxiolysis [25]; ii) IND 3 mcg/kg (Juno Pharmaceuticals or Auro Pharma) to a maximum of 200 mcg/2 mL administered as a single dose 30 minutes prior to laceration repair consistent with its onset of anxiolysis [38]; iii) Inhaled 50% N_2_O/50% O_2_ administered as needed 3 minutes prior to initial position for laceration repair without a maximum dose. Administration devices include Nitronox cylinder from Air Liquide (LHSC, London), Entenox wall mount and portable Blendox system (BC Children’s, Vancouver), Nitronox cylinder from BOMImed (CHU Sainte-Justine), Liqui-Med Analgesic Gas Mixture from Linde Canada (Stollery Children’s, Edmonton).

To minimize protocol deviations due to inconsistencies in timing of repair, such as an unexpected acute increase in the proceduralist’s clinical load, repair may be performed up to 20 minutes following INM and up to 40 minutes following IND administration and ideally up to 15 minutes of N_2_O administration to reduce AEs. The median (range) procedure duration for lacerations is 12 (1–40) minutes [25,38]. The upper limit of this procedure window is within the efficacy windows of INM [41] and IND [38]. Research nurses will administer IND and INM following a published protocol [38] using a mucosal atomizer device (MAD). MADs deliver a fine mist to the nasal mucosa for rapid administration to the systemic circulation [42]. INM or IND will be administered to each nostril, with each pair of sprays separated by ≥ 60 seconds. The maximum volume per nostril will be 0.5 mL with an extra 0.15 mL to account for dead space. N_2_O will be administered using a face mask delivering on-demand N_2_O to minimize the risk of over sedation and environmental impact and using a scavenger system to minimize exposure to the HCP. Participants will be given the option of a having a scented product applied to the inside of the mask to promote mask adherence. Research nurses will follow standard operating procedures (SOPs) in English [43] or French [44] to administer N_2_O three minutes prior to initial positioning of the participant and as needed throughout the procedure to achieve the desired level of anxiolysis, as per the treating physician. If the laceration is obscured by the facemask, it will be temporarily removed to place a suture and then re-applied, as is done in clinical practice.

### Dosing rationale

INM exhibits greater efficacy at the upper end of the dosing range (0.1–0.5 mg/kg) without serious adverse events (SAEs) [3,21]. An IND dose of 3 mcg/kg was based on a recent dose-finding study [38] which showed effective anxiolysis to 64% of children during laceration repair with 100% compliance and no adverse events (AEs) [38]. A concentration of 50% N_2_O is based on a systematic review which demonstrated that this dose was effective for laceration repair in children at least one year of age without SAEs [45–47]. Higher concentrations (70%) were associated with more AEs [30]. We chose to avoid a non-active placebo to avoid drop-ins, given that INM is the most common anxiolytic used in children for laceration repair [3].

### Co-interventions

Bedside nurses will apply LET to the wound a minimum of 30 minutes prior to suturing based on its time to peak effectiveness. Subcutaneous anesthetic may be administered by the proceduralist immediately prior to repair [48]. Any non-pharmacologic strategies and analgesics, including opioids, will be permitted prior to the interventions.

### Rescue anxiolysis/sedation

In practice, additional (rescue) anxiolysis or sedation is very rarely provided because it would impede ED flow. Instead, resistive children are typically restrained to facilitate timely repair. Nevertheless, we will record the details of additional anxiolysis/sedation provided during Phases II (preparation) or III (suturing).

### Randomization and allocation

Children will be randomized in a 1:1:1 ratio with undisclosed, varying block sizes, stratified by site. The *Maternal Infant Child Youth Research Network* (MICYRN) is a Canadian clinical research organization [49]. They will generate a randomization list stratified by site using the Research Electronic Data Capture (REDCap) platform [50] and provide it to site pharmacies who will prepare consecutively numbered intervention kits. INM and IND kits will contain drugs and MADs. The N_2_O kit will contain instructions on where to obtain it.

### Anticipated risks

SAEs (apnea, laryngospasm, clinically significant hypotension or bradycardia, airway obstruction, neurological injury, or death [51]) from single dose INM [21,22], IND [52], and N_2_O [53] are rare (0.1%) [54]. For INM, AEs are uncommon [55] but include nasal irritation and vomiting [56]. For IND, the most common AEs are transient bradycardia (2.2%), hypotension (1.2%), and oxygen desaturation (0.5%) which rarely require resuscitative interventions [27,57]. For N_2_O**,** transient AEs include nausea, agitation, vomiting, and dizziness in 4–8% [30,58]. N_2_O is a greenhouse gas and has been associated with spontaneous abortion if used for more than three hours in settings that lacked scavenging systems [59]. To minimize risks to HCPs and the amount released into the environment, we will use a scavenging system and on-demand apparatus (as opposed to free flow N_2_O). All participants will receive monitoring as per site-specific policies. These may include continuous cardiorespiratory monitoring, consisting of five-lead continuous ECG, oxygen saturation, and blood pressure, with consideration of capnography. This will commence immediately prior to administration of the intervention and continue until the participant is awake. Expected and unexpected SAEs will be reported to the Data Safety Monitoring Board (DSMB) and Health Canada, respectively. Definitions of expected and unexpected AEs can be found in Supplement 5 Unexpected Adverse Events and Definitions.

### Outcomes

All outcomes and endpoints apart from the OSBD-R will be collected by research personnel. The primary outcome is distress during laceration repair (initial positioning to tying of the last suture), as measured by the OSBD-R (scores range from 0 (no distress) to 23.5 (maximal distress)) [60–62]. The OSBD-R was chosen because: i) It measures both pain and distress and is positively correlated with self-reported pain [62]. In children, fear and pain potentiate behavioral distress [63], so a scale incorporating pain is essential; ii) It is validated for procedural pain in people 2–20 years [39,62]; iii) It is the only tool that has been used in children undergoing laceration repair [6,46,64] using video recordings to score distress [46,64] with high interrater agreement (0.9–1.0 [64]); iv) Vocalizations in any language can be used in scoring; iv) Scoring can be accomplished even if the face is obscured by an N_2_O mask [65]; v) Scores a period of observation rather than a single time point, increasing reliability [63]; vi) It correlates highly with caregiver (r = 0.38) [62] and nurse ratings of child anxiety (r = 0.73) [66], and observational fear scales (r = 0.94–0.97) [65]. The OSBD-R assesses 8 behaviors: 1) information seeking; 2) crying; 3) screaming; 4) requiring physical restraint; 5) verbal resistance; 6) requesting emotional support; 7) reaching out to be held; vocalization of pain; and 8) flailing. Based on standardized scoring [39,67], the presence or absence of each behavior is recorded every 15 seconds. A *distress score* is then calculated by summing the number of 15-second intervals during which each behavior occurred and multiplying by an expert-determined, prespecified weight from 1 to 4 based on severity of the behavior. Weightings were informed by prior studies [68–70]. There will be 4 scoring periods: Phase I (pre-intervention): 3 minutes prior to initial positioning; Phase II (preparation): initial positioning to completion of cleaning, draping, + /- infiltration of additional anesthetic; Phase III (suturing): first suture to tying knot of last suture; Phase IV (recovery): tying last knot to 3 minutes post-procedure. The weighted scores for each phase are summed and divided by the number of 15-second intervals, providing a *Total Distress Score*, which will be reported separately for each phase. Given the high frequency of scoring, research personnel will obtain a video of the participant from 3 minutes prior to 3 minutes post laceration repair using a camcorder mounted on a tripod. Once data collection is completed, the video file will be uploaded onto a shared drive. Access to the drive will be limited to outcome assessors remote from the clinical encounter who will score the OSBD-R using the videos. Two outcome assessors will score all videos of the laceration repair independently using published methods [38,69] and their *Total Distress Scores* will be averaged for analysis. We will calculate an interrater agreement (intraclass coefficient (ICC)). An external OSBD-R expert will review scores, identify any with >10% disagreement, and re-score the video for the presumed erroneous assessment. We will use a published training program for outcome assessors to optimize consistency and uniformity in scoring. It was developed by a team member (SA) and yielded an ICC of 0.88 [69]. It involves a didactic session, followed by concurrent scoring of a sample video of a child undergoing laceration repair with discussion of the meanings of distress items with an expert. Outcome assessors will then score additional sample videos until an ICC of at least 80% is achieved. Outcome assessors will be undergraduate science students without clinical experience and will be screened to rule out preconceived notions about the nature of the interventions, study objectives, and hypothesis. They will be told that “this is a study to validate a measurement tool”. They will score videos remote from the ED to avoid hearing any dialogue about the interventions. HCPs and research personnel will be asked to avoid commentaries related to effectiveness of the interventions.

Secondary outcomes include: (i) Delayed maladaptive behaviors using the PHBQ [71]) ≤ 72 hours of discharge. The PHBQ is the only scale measuring this construct and has good internal consistency (Cronbach α = 0.75–0.8) in children 2–13 years [72]; (ii) Need for additional (rescue) anxiolysis or sedation to facilitate laceration repair in phases II or III; (iii) Need for physical restraint during Phases II or III using the *Procedural Restraint Intensity in Children* (PRIC) scale. This is the only instrument measuring intensity of physical restraint. It was validated in children 2 months to 7 years undergoing venipuncture and lumbar puncture with scoring by a research nurse. Interrater and test-retest correlation was 0.98 [73]; (iv) AEs based on the Quebec Guidelines for paediatric sedation [74] and Health Canada reporting requirements [75]. AEs will be coded using the *Medical Dictionary for Regulatory Activities,* the “standardized international terminology for regulatory communication and evaluation of data pertaining to medicinal products” [76]. Research nurses will remain with participants from intervention administration until discharge to identify AEs in real time.

Additional endpoints include: i) Compliance with interventions (volume administered/prescribed; acceptance of N_2_O (yes/no)); ii) Bedside nurse, physician, caregiver, child (at least 7 years) satisfaction using a 100 mm Visual Analog Scale (VAS). The VAS has been validated for patient satisfaction in the ED [77]; iii) Nasal irritation following INM/IND sprays and N_2_O application using the *Faces, Legs, Arms, Cry, Consolability* (FLACC) scale [78]. FLACC has been validated in children during INM administration [65]. It is responsive to pain with significant differences between drug administration and baseline (p < 0.001), has high interrater agreement (ICC = 0.99) and internal consistency (Cronbach α = 0.97), and correlates highly with other paediatric pain scales [65]. FLACC is recommended by the Canadian Pediatric Society for procedural pain from 2 months to 19 years [40]; iv) Duration of procedure, post-procedure LOS, and post-intervention LOS. The latter two parameters reflect the influence of duration of sedation and onset of sedation on LOS, respectively; v) Caregiver anxiety related to laceration repair using the *State Trait Anxiety Inventory Short-5 item (STAIS-5)* measured immediately prior to Phase I. Caregiver anxiety may be an effect modifier for OSBD-R scores [79]. The STAIS-5 was highly correlated with the long version STAI-S (Cronbach α = 0.92) with excellent internal consistency (α = 0.90) [80]. Self-reported measures (PHBQ, *Gender Identity Questionnaire for Children* [81] (GIQC), and STAI-S) have been used in French [82,83]. We will collect participants’ race and ethnicity for future evidence syntheses.

Economic outcomes include: i) Number of HCPs required at the bedside during Phases II-IV for support, restraint, redirection, or care; ii) Personnel salary costs associated with time at the bedside; iii) Costs to manage AEs; iv) LOS.

### Follow-up

Research personnel will contact families 72 hours post ED discharge for late AEs [74] and delayed maladaptive behaviors using the *Post-Hospital Behavior Questionnaire* (PHBQ) [71]. Maladaptive behaviors occur up to two weeks post-sedation [84], but are most prevalent within 72 hours of sedation [11,85]. Most intervention-related AEs occur within 12 hours of discharge [23,27,51,54]. We chose 72 hours to capture most AEs yet minimize loss to follow-up. REDCap will automatically send follow-up emails to caregivers who prefer email surveys 72–96 hours and up to 14 days after discharge. If the email survey is not completed, REDCap will send up to 2 reminder emails, spaced 7 days apart. For caregivers who prefer a telephone survey, the first call will occur 72–96 hours and up to 14 days post-discharge. If the caregiver is unreachable, a minimum of 3 contact attempts must be made, with attempts spaced at least 7 days apart. If the follow-up period includes a weekend or holiday, and the initial call is delayed, we will ensure that a minimum of 3 attempts are made once research personnel is available. 3. If a survey is missed, or reminders have been sent by REDCap, but the survey is still incomplete, research personnel will follow up via telephone between REDCap’s automatic reminders.

### Patient engagement

Our institution’s Family Advisory Council, our patient partner (CH), and our steering committee informed the trial’s eligibility criteria, burden of interventions, and outcomes. From their experiences as caregivers of children who visited a health care setting, they reviewed and provided feedback on the content of the recruitment pitch, letters of information, consent, and assent. Results will not be directly disseminated to participants but will be provided upon request.

### Sample size

We used the Average Length Criterion (ALC) [86], a Bayesian method for sample size estimation. The ALC ensures 95% coverage for mean *Total Distress Score* in each arm. The ALC controls the precision of the efficacy estimate by selecting the sample size so that the posterior credible interval (analogous to a confidence interval) for mean *Total Distress Score* is below a pre-specified threshold. Based on simulations, a threshold of 0.67 resulted in a 10-fold increase in precision compared to the distribution of *Total Distress Score* from previous studies (priors) and yielded a sample size of N = 100/arm. There is no standard of care so the adaptive design will rank the three interventions based on efficacy. We will declare futility and superiority based on the probability that an intervention is superior (P_*best*_) based on the primary outcome. At the interim futility analysis (N = 53/arm; adjusted for dropouts), an intervention will be considered futile and dropped if P_*best*_ is < 0.026. At the final analysis, an intervention will be considered superior if P_*best*_ is > 0.975. These thresholds were chosen to ensure a type I error <2.5%. Sample sizes with 0, 1, and 2 arms dropped are 315, 263, and 159, respectively and account for 5% dropouts. Previous studies using the OSBD-R in children undergoing distressing procedures estimated minimal clinically important differences (MCID) of 1.05–2 [46,87–90]. We tested the power of our approach using three values for the true OSBD-R difference between the interventions, 0.5, 1.0 and 1.5, with a simulation study comparing two approaches for ranking interventions for a comparative effectiveness trial. We found that P_*best*_ resulted in greater power than alternative Bayesian methods at 80% [91]. We have not adjusted for within site clustering because each site will have at least 50 proceduralists (physicians, nurse practitioners, and trainees); each performing a small number of laceration repairs, minimizing the effect of clustering. Therefore, we will account for site as a random effect for greater power in our adjusted analysis.

### Analysis

The primary analysis will be conducted by intention-to-treat. We will average the *Total Distress Scores* across phases II and III of the laceration repairs, for our computation of the posterior score distributions. The primary analysis will adjust for age, sex, gender/gender-expression, pre-intervention analgesia, caregiver STAI-5, physician’s experience suturing lacertions (years) as fixed effects, and site as a random effect. The mean difference in *Total Distress Score* [92], as well as odds ratios for AEs, PHBQ, and PRIC scores will be estimated using Bayesian models and we will report posterior means, SDs, and 95% high density posterior credible intervals. Additional anxiolysis or sedation is very rare, if ever provided in practice, so will be presented with descriptive statistics alone. We will report severity, frequency, duration, and relationship of AEs to intervention and sex given the shorter duration of action of midazolam [93] and N_2_O [94] in females. We will use a prior for *Total Distress Score* for each intervention, informed by our IND dose-finding trial [38] and literature [46,95]. We will use predefined minimally informative priors for other analyses [96,97] along with descriptive statistics.

Missing data is expected to be minimal as the primary outcome will be collected in the ED. If missing data is less than or equal to 5%, we will use a complete case analysis. If greater than 5% and baseline characteristics differ substantially, we will use a joint Bayesian model for missingness and outcomes.

In terms of frequency of analyses, an interim analysis for futility will occur at N = 53/arm. An intervention will be considered futile and dropped only if there is a < 2.6% chance it is the best intervention. This threshold yielded a type I error rate of <2.5% from 10,000 simulations [91]. The overall power is 80%, even if an intervention is dropped. The Data Safety Monitoring Board (DSMB) will review AEs biannually and as needed to stop the trial for safety if there are more than the expected number of AEs and SAEs based on the literature [22,27,30]. We are using a Bayesian analysis so stopping criteria do not impact the primary analysis.

For *Total Distress Score*, we will conduct subgroup analyses using a Bayesian linear model with a test of interaction between group and: i) sex; ii) gender (masculine versus feminine versus non-binary) and gender-expression (trans- versus cis-gendered). Female sex and feminine gender have been associated with greater pain sensitivity [98] so we hypothesize that OSBD-R and pain scores may be higher in these groups. These are exploratory analyses so sample size will not be adjusted. Young children may not express gender identity so caregivers will be asked to identify “gender expression” using the caregiver-reported, *Gender Identity Questionnaire for Children* [81] (GIQC). The GIQC had a sensitivity of 87% among children 2–12 years referred to a gender-identity clinic and a specificity of 95% [81]. We will report sex and gender-based analyses using the *Sex and Gender Equity in Research Guidelines* [99]. We will use the *Instrument for assessing the Credibility of Effect Modification Analyses* to assess the credibility of potential effect modifiers [100].

We will conduct an economic analysis to weigh the cost effectiveness of the interventions. Quality Adjusted Life Years are less able to inform the effectiveness of short-term interventions so we will use an individual-level state transition model involving pre-procedure, procedure, post-procedure, and discharge. The model incorporates *Total Distress Score,* LOS, HCP (including research nurse/CCLS) salary costs for time spent at the bedside, and costs to manage AEs. This model assumes that *Total Distress Score* and AEs influence LOS. We developed this approach using analogous data from the literature and found a robust model could be generated [101]. We will extract the costs for each site and estimate the individual cost by multiplying the number of minutes and personnel cost for each state, followed by adding the cost of drugs. Power calculations were not performed because the Bayesian approach asserts that the optimal agent should be selected based on its “mean net benefits”, even if statistical significance is not reached [102]. The social demographics of each site are reflected in the model as site is a random effect. Costs will be adjusted for inflation. For stakeholders accustomed to incremental cost-effectiveness ratios, we will report comparison of total costs to *Total Distress Score* [103] and follow the 2022 *Consolidated Health Economic Evaluation Reporting Standards* [104].

### Data Management

Data management services will be provided by MICYRN. Study data will be entered and managed using REDCap tools hosted and supported by MICYRN. Data will be stored and shared according to the FAIR principles (findable, accessible, interoperable, reusable) using Scholarship@Western, a publicly accessible repository. Data will be entered directly into the study database using a WIFI enabled encrypted iPad. In the case of a technical failure, data will be collected on paper and then transcribed into REDCap by the research nurse or site coordinator. The study participant’s contact information will be securely stored at each clinical site for internal use during the study. At the end of the study, all records will continue to be kept in a secure location for 15 years, as per Health Canada requirements. Individual participants and their research data will be identified by a unique study identification number.

### Monitoring

Monitoring for data quality and regulatory compliance will be performed by the University of Alberta’s Clinical Trials Office. The Office is an independent unit housed within the university’s central administration that provides arms-length review of all University of Alberta sponsored trials, at least three times per year. Details of clinical site monitoring will be documented in a Clinical Monitoring Plan. This trial will also be monitored federally by Health Canada (approval number HC6–024-c267577) and at the institutional level by site-specific clinical research oversight bodies.

Safety oversight will be under the direction of the DSMB which will function independently of the investigators. The DSMB is independent from the steering committee and includes an investigator with multicenter research experience, a knowledge user with laceration repair experience, and a senior clinical trialist as Chair. The DSMB will have open and closed meetings and operate under an approved charter. The research team will meet with the DSMB at baseline and bi-annually for recommendations. The DSMBs will review descriptive statistics of AEs biannually and as needed to stop the trial for safety if there are an unacceptably high number of SAEs (more than expected from the literature). At the DSMB’s request, they can receive posterior credible intervals or predictive probabilities. The decision to stop the trial for safety reasons will be left to the discretion of the DSMB.

### Timeline

Enrollment was launched at the lead site on December 19, 2023. Training and subsite approvals will take three months. Recruitment is expected to take 24 months. Data verification and analysis will occur within three months of completion. KM and QI activities will occur within 12 and 24 months of completion, respectively. The study duration is 54 months.

### Ethics

Written ethics approval at the lead site was obtained from Western University’s Health Sciences Research Ethics Board. The other participating sites will acquire local ethics approval prior to enrollment. All protocol amendments will be submitted for approval to Health Canada before being communicated to each site. All protocol amendments will be added to the clinicaltrials.gov registration and implemented only after Health Canada and REB approval. All study participants, or their caregivers, will be notified if any new findings become available which may be in the best medical interest of the study participant or impact their willingness to continue participation in the study.

### Access to data

The principal investigator will have access to the final trial dataset. The data safety monitoring board will have access to safety data throughout the trial upon their request. Data can be provided to other parties upon written consent approved by the principal investigator. There are no plans for granting public access to the participant-level dataset or statistical code.

### Sub-studies

Depending on data integrity, the following sub-studies are planned:

i) Risk factors for delayed maladaptive behaviors
ii) Concordance of the OSBD-R with pain scales and sedation scales
iii)  Relationship between sex, gender, gender-identity, race, and ethnicity on procedural distress
iv) Relationship between caregiver anxiety and procedural distress

## Discussion

### Limitations

Comparative effectiveness trials with fixed allocation often fail to produce statistically significant results and require large sample sizes to detect differences [105]. Our Bayesian adaptive design [106] will calculate the probability that an anxiolytic is superior with fewer participants, greater power, and demonstrate their relative benefits. [107] Rather than relying on effect estimates alone, HCPs may use a ranking of efficacy to inform their anxiolytic choice. Despite this adaptation to a traditional frequentist approach, there are several limitations.

Compliance with drug administration may be an issue, particularly in younger children. Nevertheless, young children are also more likely to resist laceration repair, making it necessary to determine effectiveness in this age group. Specifically, several authors have suggested that N_2_O’s benefit is greater in older children because they cooperate with a face mask and reduce entrainment of room air [58,108]. However, a 2023 systematic review [30] identified three trials where 50% N_2_O delivered by continuous flow face mask, was effective in children under 3 years [45–47], suggesting even young children are compliant. Conversely, up to 40% of children experience discomfort with administration of INM [22]. In our trial, INM will be administered using at most, a single pair of sprays with the assistance of a certified child life specialist or caregiver, making non-compliance less likely. Poor compliance has not been reported with IND. In fact, an dose-finding trial found that all 55 children aged 1–10 years accepted the IND dose [38]. To understand whether efficacy is influenced by compliance in standard practice, we will measure the proportion of dose accepted, discomfort with IND and INM administration, and N_2_O mask acceptance.

For most outcomes, loss to follow-up is expected to be low because they will be collected in the ED. However, missing data related to caregiver and child satisfaction, 72-hour AEs, and PHBQ scores are possible. In our IND dose-finding [38], 100% of participants completed the 72-hour follow-up. We will use successful strategies to minimize loss to follow-up and missing data, including collecting more than one phone/email contact, a phone/email reminder at 24–48 hours post-discharge, and reviewing the medical record for return visits.

### Dissemination

We will be supported by: Western University’s *Knowledge Exchange, Impact, and Equity Diversity Inclusivity and Decolonization (EDID) in Research*; and We will disseminate findings using the *Solutions for Kids in Pain (SKIP)’s* [109] website, social media, and partner blogs; and *Children’s Healthcare Canada’s* [110] national webinars. Given that more than 80% of children are managed in community EDs, we will use the knowledge translation platforms of *Translating Emergency Knowledge for Kids (TREKK)* [111] and integrate our findings into a “Bottom Line Recommendation”. We will present our work at continuing medical education events, network meetings, and societal conferences. We will collaborate with a data visualization specialist to help create text-minimized and gender-inclusive infographics in English and French. We will submit our protocol, design, and analysis in open-access journals and findings in a high-impact journal. Byline authorship will be offered to co-investigators who meet International Committee of Medical Journal of Editors requirements. We will upload publications and other KM materials on Scholarship@Western, an open access digital repository and publicize them using Western Media Relations. Key messages to knowledge users are to consider anxiolytics for laceration repair (HCPs), anxiolytics may be necessary and are safe (families), the optimal anxiolytic is cost-effective (policy makers), and adaptive designs can improve trial efficiency (researchers).

### Amendments

Amendments to the study protocol will be submitted to Health Canada. Once a Health Canada No Objection Letter has been received, sub-sites will be notified by email and sent the updated protocol. Each site will be responsible for seeking approval of the amended protocol through their office of research ethics. Termination of the study will follow the same approach in consultation with the DSMB.

## Supporting information

S1 File SPIRIT 2013 Checklist. (DOCX)

S2 File SPIRIT Figure. Schedule of enrollment, interventions, and assessments.
(DOCX)

S3 File Ethics Approved Protocol. (DOCX)

S4 File Informed Consent and Assent Forms. (DOCX)

S5 File Unexpected Adverse Events and Definitions. (DOCX)

S6 File Edmonton Ethics Approval. (PDF)

S7 File Montreal Ethics Approval. (PDF)

S8 File Vancouver Ethics Approval. (PDF)

S9 File London Ethics Approval. (PDF)
