# Supplementary material for: Anxiolysis for laceration repair in children: study protocol for an open-label multicenter adaptive trial (ALICE)
Source: PLoS One. 2025 Jun 4;20(6):e0324515. doi: 10.1371/journal.pone.0324515 (PMC12136299; doi:10.1371/journal.pone.0324515)
Supplement: S2 File — Schedule of enrollment, interventions, and assessments. (DOCX) [file pone.0324515.s002.docx]

S2 SPIRIT Schedule. SPIRIT Schedule of enrolment, interventions, and assessments.

|  | **STUDY PERIOD** | | | | | | | |
| --- | --- | --- | --- | --- | --- | --- | --- | --- |
|  | **Enrolment** | **Allocation** | **Post-allocation** | | | | | **Close-out** |
| **TIMEPOINT**** | ***-t_1_*** | **0** | ***t_1_^1^*** | ***t_2_^2^*** | ***t_3_*** | ***t_4_*** | ***etc.*** | ***t_x_*** |
| **ENROLMENT:** |  |  |  |  |  |  |  |  |
| **Eligibility screen** | X |  |  |  |  |  |  |  |
| **Informed consent** | X |  |  |  |  |  |  |  |
|  |  |  |  |  |  |  |  |  |
| **Allocation** |  | X |  |  |  |  |  |  |
| **INTERVENTIONS:** |  |  |  |  |  |  |  |  |
| ***Intranasal midazolam*** |  |  | X |  |  |  |  |  |
| ***Intranasal dexmedetomidine*** |  |  | X |  |  |  |  |  |
| ***Inhaled nitrous oxide*** |  |  |  |  |  |  |  |  |
| **ASSESSMENTS:** |  |  |  |  |  |  |  |  |
| ***Anxiolysis***  ***(OSBD-R)*** |  |  | X |  |  |  |  |  |
| ***Maladaptive behaviors*** |  |  |  | X |  |  |  |  |
| ***Additional Anxiolysis*** |  |  | X |  |  |  |  |  |
| ***Adverse Events*** |  |  | X | X |  |  |  |  |
| ***Physical Restraint*** |  |  | X |  |  |  |  |  |
| ***Compliance with Administration*** |  |  | X |  |  |  |  |  |
| ***User Satisfaction*** |  |  | X |  |  |  |  |  |
| ***Caregiver Anxiety*** |  |  | X |  |  |  |  |  |
| ***Number of Providers at Bedside*** |  |  | X |  |  |  |  |  |

^1^Refers to during laceration repair

^2^Refers to within 72 hours of laceration repair
