# Supplementary material for: Anxiolysis for laceration repair in children: study protocol for an open-label multicenter adaptive trial (ALICE)
Source: PLoS One. 2025 Jun 4;20(6):e0324515. doi: 10.1371/journal.pone.0324515 (PMC12136299; doi:10.1371/journal.pone.0324515)
Supplement: S3 File — (DOCX) [file pone.0324515.s003.docx]

Anxiolysis for laceration repair in children: An open-label multicenter adaptive trial (ALICE)

**Short Title: ALICE**

**Trial registration:**

Clinicaltrials.gov Identified Number: NCT05383495

Protocol number: ALICE2022

**Protocol Version Number:** V 3.2, 2025-02-12

**Funding:**

Western Strategic Support (WSS) and Academic Medical Organization of Southwestern Ontario (AMOSO). The funders have no role or authority in study design, collection, management, analysis, and interpretation of data, writing of the report, or the decision to submit the report for publication.

**Study Sponsor:**

Naveen Poonai, MSc MD FRCPC

Associate Professor of Pediatrics, Epidemiology & Biostatistics, and Internal Medicine

Schulich School of Medicine & Dentistry, Western University

Chair and Scientist, Children’s Health & Therapeutics Division, Children’s Health Research Institute

Research Director, Division of Paediatric Emergency Medicine

800 Commissioners Road East, Room E1-106, London, Ontario, N6A 5W9

**Qualified Principal Investigator:**

Naveen Poonai MSc MD

**Co-Investigators (Name and Department):**

Vikram Sabhaney, MD, Assistant Professor, Paediatrics, University of British Columbia

Samina Ali, MDCM, Professor, Paediatrics, University of Alberta

Maala Bhatt, MD, Associate Professor, Paediatrics, University of Ottawa

Evelyne Doyon-Trottier, MD, Associate Professor, Paediatrics, Universite de Montreal

Anna Heath, PhD, The Hospital for Sick Children, Assistant Professor

Nam Anh Tran, PhD, The Hospital for Sick Children

Jennifer Turnbull, MD, Associate Professor, Paediatrics, McGill University

Martin Osmond, MD, Professor, Paediatrics, University of Ottawa

Vinolia Arthur-Hayward, Clinical Research Coordinator, London Health Sciences Centre

**Collaborators:**

Kyna Patterson, Certified Child Life Specialist, London Health Sciences Centre

Raju Polacherla, MD, Associate Professor, Schulich School of Medicine & Dentistry

**Roles and Responsibilities:**

Dr. Poonai is the lead investigator responsible for trial oversight. Vinolia Arthur-Hayward is the project coordinator. Dr. Heath is the biostatistician and methodologist. All other investigators are knowledge experts that will contribute to the design, protocol, results interpretation, and manuscript preparation.

ALICE: Protocol Signature Page

| Study Title: | Anxiolysis for laceration repair in children: An open-label multicenter adaptive trial (ALICE). |
| --- | --- |
| REB# or CTO# | REB #120985 |
| Sponsor: | Lawson Health Research Institute and Western University |
| Principal investigator: | Dr. Naveen Poonai |
| Protocol version: | Version 3.2 |
| Protocol version date: | 12Feb2025 |

I, the undersigned, have read and understood the protocol specified above and agree to its content. I agree to perform and conduct the study as described in the protocol and in accordance with the relevant regulations and standards outlined in the protocol.

# Principal Investigator Signature:

| Dr. Naveen Poonai  _______________________ |  | _14/__Feb_/__2025____ |
| --- | --- | --- |
| Name of Principal Investigator | Signature | Date  (dd/mmm/yyyy) |

# Site Qualified Investigator Signature:

Site Name:

| _______________________ |  | _____/_____/______ |
| --- | --- | --- |
| Name of site Qualified Investigator | Signature | Date  (dd/mmm/yyyy) |

Please save as a PDF version and add copy to your TMF Binder

**Table of Contents**

| Introduction | 4 |
| --- | --- |
| 1. Background and rationale | 4 |
| - 1. Clinical Impact | 5 |
| 1. Objectives | 5 |
| 1. Trail Design | 6 |
| Methodology: Participants, interventions, and outcomes | 6 |
| 1. Study Setting | 6 |
| 1. Eligibility Criteria | 6 |
| - 1. Inclusion Criteria | 6 |
| - 1. Exclusion Criteria | 7 |
| 1. Intervention | 7 |
| - 1. Following intervention administration | 9 |
| - 1. Adherence to protocol | 10 |
| - 1. Concomitant Therapy | 10 |
| - - 1. Co-intervention | 10 |
| - 1. Discontinuation of study intervention | 11 |
| - 1. Rescue Medication | 11 |
| 1. Outcomes | 12 |
| - 1. Secondary Outcomes | 13 |
| - 1. Other endpoints | 13 |
| 1. Enrollment Timeline | 14 |
| 1. Sample Size | 15 |
| 1. Recruitment | 15 |
| Methods: Assignment of intervention | 15 |
| 1. Allocation/ Sequence generation/ Allocation concealment mechanism | 15 |
| 1. Blinding | 15 |
| - 1. Unblinding procedures | 16 |
| Methods: Data collection, Management, and Analysis | 16 |
| 1. Data Collection | 16 |
| 1. Data management | 17 |
| 1. Statistical Methods | 18 |
| Methods: Monitoring | 20 |
| 1. Data Monitoring | 20 |
| - 1. Harms (Study Assessment and Procedures Assessment of Safety) | 21 |
| - 1. Reporting AEs, Serious Adverse Events and Unexpected Drug Reactions | 22 |
| - 1. Monitoring and Audit | 24 |
| Ethics and Dissemination | 25 |
| 1. Research ethics approval | 25 |
| 1. Protocol amendments | 25 |
| 1. Consent or Assent | 25 |
| 1. Confidentiality | 26 |
| 1. Declaration of interests | 26 |
| 1. Access to data | 26 |
| 1. Ancillary and post-trial care | 27 |
| 1. Dissemination policy | 27 |
| 1. References | 28 |
| - 1. Appendix | 32 |
| - 1. Appendix I: Letter of Information and Consent form | 32 |
| - 1. Appendix II: Assent form | 32 |
| - 1. Appendix III: Unexpected Adverse Events | 33 |

**Introduction**

1. **Background and Rationale**

Accounting for almost half of all procedures performed, lacerations are the most common traumatic injury reason for an ED visit in children (1). There is compelling evidence that children experience considerable distress during laceration repair (2-4), despite routine application of a topical anesthetic, lidocaine-epinephrine-tetracaine (LET) (5, 6) or injected lidocaine. Certified child life specialists (CCLSs) can help children cope with distress (2) but are not widely available (7) outside of and even within some paediatric centres. Untreated distress in childhood can lead to anxiety, needle phobia, and fear of medical care as adults (8). The *American Academy of Pediatrics (9), Canadian Paediatric Society (10),* and *American Academy of Pediatric Dentistry* strongly recommend minimizing discomfort and controlling behavior and movement during procedures but there is little specific guidance on laceration repair (11). A recent national survey of Canadian paediatric ED physicians found that 86% believed that at least a quarter of young children experienced distress during laceration repair that was significant enough to require physical restraint (3). Pharmacologic anxiolysis may be mitigate the need to forcefully restrain a child. However, evidence for the most effective agent is lacking. Although anxiolytics such as midazolam and nitrous oxide (N_2_O) are frequently used (6), there are drawbacks including variable efficacy and nasal irritation in the case of intranasal (IN) midazolam (12) and lack of cooperation in younger children in the case of N_2_O (13). IN dexmedetomidine is a potentially effective anxiolytic for laceration repair that could overcome these limitations.

**IN dexmedetomidine** is a relatively new anxiolytic with sedative and analgesic properties (14). It is tasteless, non-irritative, and highly concentrated (100 mcg/mL). A maximum dose of 200 mcg/2 mL can be given with 2 pairs of 0.5 mL IN sprays. Our team recently published a systematic review of IN dexmedetomidine for distressing procedures, and demonstrated that it was well tolerated by more than 90% of children and provided adequate anxiolysis in more children (79%) versus conventional anxiolytics (midazolam, chloral hydrate) (60%) (15). Only one trial investigated IN dexmedetomidine in children for laceration repair and 70% were deemed “not anxious” compared with 100% for IN midazolam (11%) (16). However, the study focused on initial positioning rather than during repair, where the two options performed similarly. For proof of concept and to identify the optimal dose and feasibility of IN dexmedetomidine, we recently completed a pilot of 55 children undergoing laceration repair (17). All children tolerated IN administration, the consent rate was 82%, and there were no serious adverse events (AE). A dose of 3 mcg/kg balanced efficacy with duration of sedation, in line with others studies’ findings (6, 18).

Prior to the availability of IN midazolam, oral midazolam was the most commonly used anxiolytic for laceration repair in children (6). However, onset and duration of action and efficacy can be unreliable and its bitter taste leads to poor compliance (12). **IN midazolam** using a mucosal atomizer device (MAD) (Figure 1) is increasingly popular (3) because it overcomes these limitations. Compared to oral midazolam, IN midazolam has a shorter onset of action (28 vs 34 minutes) (19, 20) but causes nasal irritation in up to 40% of patients (19, 21). In addition, IN midazolam has variable efficacy for laceration repair (19, 20) and more adverse effects compared to nitrous oxide or IN dexmedetomidine (16, 22). The largest study of IN midazolam was a retrospective study of combination IN midazolam plus IN fentanyl. A lower dose of midazolam (0.2 mg/kg) resulted in fewer adverse effects (0.7%) than with higher doses (2-5%) (16, 19). Adding IN fentanyl provided superior efficacy where only 2.4% of patients experienced a treatment failure (23).

The second most common anxiolytic is **inhaled N_2_O** (6), a tasteless gas that is administered in a concentration of 30-70% with oxygen (24). N_2_O is safe (25) and effective (13, 24) for minor painful procedures. Onset of effect is rapid with a peak effect in 3-5 minutes (26), and effects dissipate quickly (3-5 minutes) upon discontinuation (27, 28). Minor adverse effects, such as dizziness, occur in 8% of patients (24). However, based on the United Kingdom National Clinical Guideline Centre, it’s unclear whether uncooperative children will comply with N_2_O administration (13). Our recently completed systematic review of 29 trials of N_2_O for painful procedures in children (29) found 50% N_2_O was superior to subcutaneous lidocaine (30), placebo (31, 32), and oral midazolam (22). Only 5 trials focused on laceration repair (22, 30-33) but they were small and limited by non-validated or lack of measures of anxiety (30-33).

Despite frequent use of anxiolytics such as midazolam and N_2_O (3), our national survey found that 80% of Canadian paediatric ED physicians strongly believed that “children experience significant enough distress to warrant anxiolysis” and it would “improve the patient care experience” and that use of anxiolysis “improve the patient care experience”, with frequent use of anxiolytics such as midazolam and N_2_O in practice (3). To improve care, a rigorous trial of the novel anxiolytic, IN dexmedetomidine, and further research on two frequently used anxiolytics, IN midazolam and N_2_O, with dissemination of our findings, is urgently needed.

- 1. **Clinical Impact**

In the short-term, our work will lead to a less distressing experience for children. Administration of IN dexmedetomidine is well tolerated by children (15) as is the administration of N_2_O and falls within nurses’ skill set as Regulated Health Professionals (34, 35). Anxiolysis may also reduce the need to redirect and restrain a child (22), avoiding the psychological trauma or physical restraint and reducing the need for an extra HCP (e.g. another nurse or patient safety aid). As most children with lacerations are seen outside of tertiary care centres, disseminating our results to community EDs on how to administer IN and inhaled anxiolysis safely and effectively will have a broad, longer-term impact beyond our trial participants. A less distressing experience for children will improve parental satisfaction and this correlates highly with perceptions of the healthcare experience (36) and is an important determinant of compliance with discharge instructions (37), such as guidance to return to the ED for wound complications. Procedural distress is often difficult to manage in children because most lacerations occur on the face (23), making distraction difficult. Untreated distress can result in poor appetite and sleep, fear of future medical care, and chronic pain (38). During laceration repair, many children are physically restrained by multiple nurses or patient safety aids while the clinician hastily tries to complete the procedure. Parents are significantly affected by their child’s discomfort and look to providers to relieve distress (36). A more relaxed child will reduce the time to conduct the procedure, reduce the risk of a heightened response to future medical care, and improve parental perceptions of their child’s healthcare experience (37). It may also decrease the need for physical restraint (22) and promote more efficient use of nurses’ or patient safety aids’ time. To maximize the impact of our work, we will translate our findings beyond our tertiary care centre to community EDs, where most children are seen. To produce **sustainable change,** we will engage these centres at trial design and involve them in the development of a **practice guideline for anxiolysis in laceration repair**, which will be disseminated through in-service workshops and evaluated using clinical informatics.

**2.0 Objectives**

To determine which anxiolytic results in lower behavioral distress during laceration repair, IN dexmedetomidine, IN midazolam, or inhaled N_2_O. Comparing anxiolytics that are familiar to clinicians in a large clinical trial is essential to effective knowledge translation. In fact, our national survey found that 64% of Canadian paediatric ED physicians would consider IN dexmedetomidine if there was evidence of benefit over commonly used agents (3).

**3.0 Design**

Phase III, open-label, multicentre, adaptive trial.

**Methodology: Participant, Interventions and outcomes**

**4.0 Setting**

Participants will be recruited from the EDs at 4 participating PERC sites (LHSC, London; CHU Ste Justine, Montreal; BC Children’s, Vancouver; Stollery Children’s, Edmonton)

**5.0 Eligibility**

Criteria reflect the epidemiology of laceration injuries in children (39, 40) and the age range that the Observational Scale of Behavioral Distress-Revised (OSBD-R) (41), the primary outcome, has been validated. They were also informed by our clinical experts with the interventions, CCLS, and patient partner. We chose not to restrict the length or location of lacerations to optimize externally generalizability. These include single lacerations requiring interrupted sutures alone because they are the most common where repair can typically be performed in < 20 minutes (39) and analgesia with local anesthetic (versus regional or general anesthesia) is widely practiced. However, we will exclude a) lacerations requiring closure of muscle or fascial layers because they are less common and take longer to complete, thereby requiring additional doses of anxiolytics, analgesics, or sedatives and b) lacerations where the physician uses dermal adhesives rather than sutures (42, 43) because repair can often be accomplished in < 5 minutes and with non-pharmacologic therapies alone to reduce anxiety.

**5.1 Inclusion Criteria**

1. Age 2-12.99 years
2. Signed informed consent
3. Single or multiple lacerations no more than 2 cm apart for which the treating physician believes repair does not require IV sedation
4. No concomitant fracture or dislocation.
5. Repair to involve sutures and performed by the emergency physician or their designate (any HCP authorized to perform laceration repair)
6. The child or caregiver desires anxiolysis for laceration repair
7. Predicted to resist positioning for repair by the parent, child life specialist, bedside nurse, or physician.
8. Local anesthesia and or topical lidocaine-epinephrine-tetracaine (LET) +/-r infiltrated lidocaine.

**5.2 Exclusion Criteria**

1. Concomitant fracture or dislocation or repair (concomitant nailbed injury requiring management).
2. Received any opioid, sedative, or anxiolytic medication in the ED prior to the procedure.
3. History of hypersensitivity to dexmedetomidine, midazolam, or nitrous oxide.
4. Occlusion of at least one nostril due to polyps or septal deviation.
5. Administration of an α2-adrenergic receptor agonist (eg. clonidine), sedative, or anxiolytic < 24 hours prior to intervention.
6. Bradycardia or hypotension below 2 standard deviations (SD) of age-related normal value.
7. Pre-existing renal insufficiency, uncorrected mineralocorticoid deficiency, pulmonary hypertension, uncorrected cyanotic congenital heart disease, cardiac conduction disorder, pulmonary edema, vitamin B12 or folate deficiency or disorders, phenylketonuria, or psychotic disorder.
8. Impaired level of consciousness or respiratory instability.
9. Conditions associated with potential accumulation of gas within body spaces (e.g. pneumothorax, pneumoperitoneum),
10. Suspected pregnancy (as reported by the patient) or confirmed pregnancy
11. Inability to vocalize pain due to motor deficits
12. Unable to comprehend study tasks in English or French in the absence of a native language interpreter
13. Weight > 65 kg to avoid underdosing of intranasal intervention.
14. Lacerations requiring closure of muscle or fascial layers.
15. Lacerations where the physician uses dermal adhesives rather than sutures.
16. *Inability to use the* Observational Scale of Behavioral Distress – Revised *(OBSD-R) due to a motor deficit*
17. American Society of Anesthesiologists (ASA) classification >II

Note American Society of Anesthesiologists class I (healthy) or II (controlled mild to moderate systemic disease).

NB: Fasting status will not be a consideration based on recent sedation guidelines (44)

**6.0 Interventions**

Eligible participants will be randomly allocated in a 1:1:1 ratio with varying block sizes to either:

a) IND (Juno Pharmaceuticals or Auro pharma) 3 mcg/kg [100 mcg/mL (maximum 200 mcg or 2 mL)] OR

b) INM (Sandoz or Pfizer) 0.4 mg/kg [5 mg/mL (maximum 10 mg or 2 mL)] OR

c) 50% nitrous oxide in 50% oxygen using a Nitronox^TM^ cylinder Air Liquide (LHSC, London), Entenox wall mount and portable Blendox system (BC Children’s, Vancouver), Nitronox cylinder (BOMImed, CHU Sainte-Justine), Liqui-Med Analgesic Gas Mixture (Linde Canada, Stollery Children’s, Edmonton) using a facemask for free-flow gas delivery for on-demand delivery to the procedure physician’s desired level of anxiolysis or light sedation.

Due to procurement differences at the participating clinical trial sites, brands of study IP to be used in the trial may differ from what was submitted in the original CTA. In this case, all products will be equivalent and sourced from the Canadian market.

IND and INM will be administered 30 minutes and 15 minutes prior to the start of Phase I (pre-preparation), respectively, consistent with their times to peak efficacy. Phase I or preparation is the initial positioning to completion of irrigation, cleaning with sterile solution, draping, +/- infiltration of additional anesthetic. Both agents will be administered after LET is applied. Guided by our published protocol for the *Ketodex* trial, the child will be reclined to 45^o^ on the bed or caregiver’s chest in a supine position (Figure 1) (45). Using a MAD, the research nurse will administer INM or IND to each nostril, with each pair of sprays separated by > 60 seconds and a maximum time of 3 minutes between sprays. The maximum volume per nostril will be 0.5 mL with an extra 0.15 mL to account for dead space. The maximum number of pairs of sprays is 2. In our pilot study, 2 pairs of 0.5 mL sprays were well accepted by all 55 participants (19, 20, 46).

Guided by our clinical experts in N_2_O and their SOPs, N_2_O will be administered starting 3 minutes prior to the start of Phase I (pre-preparation) and then administered as needed throughout the procedure to achieve the desired level of anxiolysis, as per the treating physician, using a facemask (Figure 2).

**
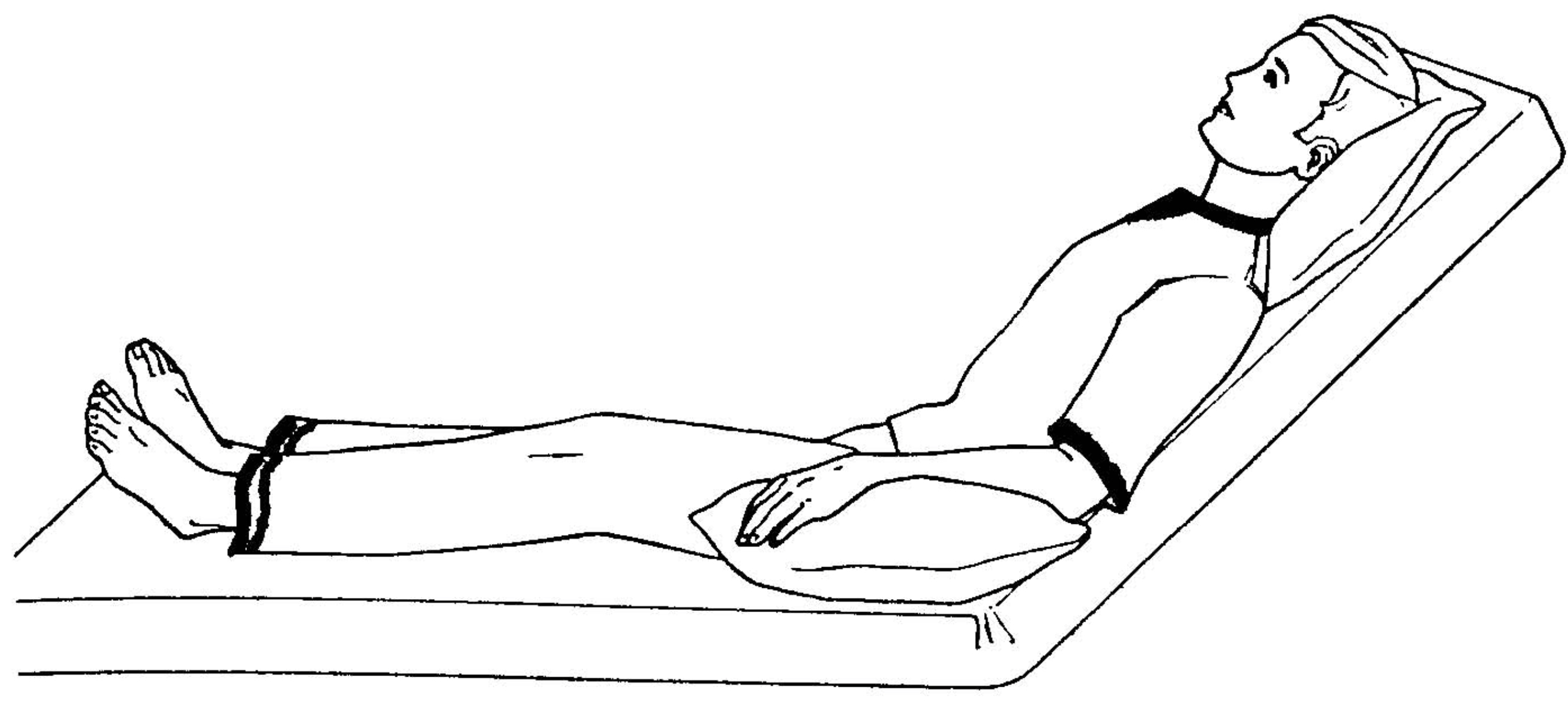
**

**Figure 1. Intranasal intervention administration**

In order to administer N_2_O, a NITRONOX™, Entenox wall mount Blendox, Liqui-Med Analgesic Gas Mixture (50:50 mix) cylinder will be used. The physician, RT or nurse must ensure the proper functioning of the demand valve and follow the steps below:

- Open the cylinder to verify the quantity of available gas (minimal quantity is at least 720 pound per square inch [PSI]).
- Connect the grey scavenger system hose to the yellow (“vacuum”) wall suction.
- May need to use the bleed button to trigger demand valve an ensure proper functioning.
- Ensure that the mixture reading (middle readout) is in the “green” zone.

During sedation the physician, RT or nurse will:

- Provide optional choice of a scented lip balm product to promote facemask acceptance. This will be applied on the inside of the facemask by the research team member.
- Apply the facemask while ensuring that the nose and mouth are covered to maintain a good seal. If there is not a good seal or if the patient does not breathe deeply enough, then the patient will not be able to trigger the valve for delivery of the N_2_O. The health care professional may assist by holding the mask if needed for younger or less cooperative children. Older or more cooperative children may hold the mask themselves. Adding or removing air may be required through the mask’s Luer lock. Caregivers may also assist by either by holding the mask or by holding the child. The mask can be temporarily removed to facilitate suture placement if the laceration is obscured by the mask; given that N_2_0 has a 2–3 minute duration of action, the child should remain sedated during this brief removal of the mask.
- If nausea or other side effects occur, the mask can be removed temporarily, then re-administered.
- Instruct the participant to inhale through the mask as if taking a deep breath through a straw.

**6.1 Following intervention administration:**

- Ensure that the patient’s level of consciousness has returned to baseline
- Monitor vital signs as per site-specific institutional requirements
- Document any adverse effects and interventions required
- Document total time of N_2_O administration (if applicable)
- Clean and replace N_2_O equipment as necessary:
  - Disinfect the machine with disinfectant wipes such as Accel Prevention® after use as per standard wiping protocol
  - The mask and circuit must be discarded between each patient.


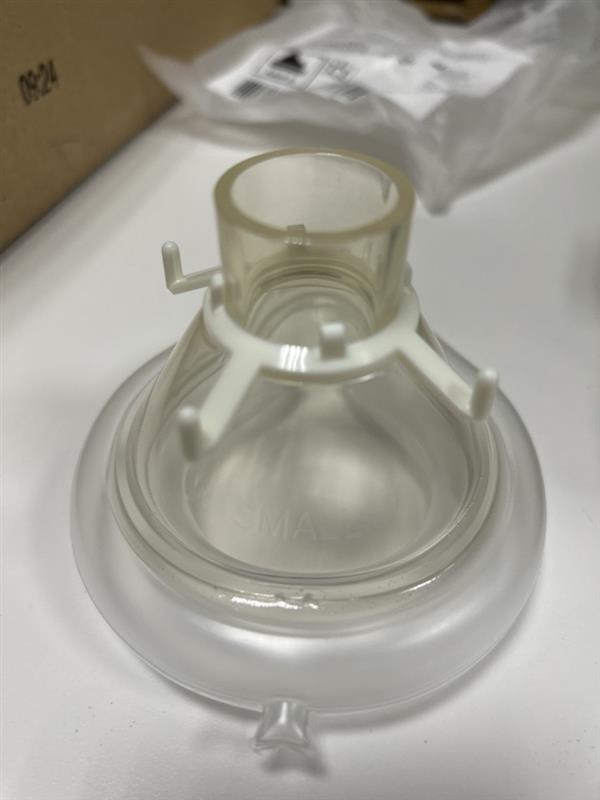


**Figure 2. Facemask for nitrous oxide administration**

**6.2 Adherence to Protocol**

The allocation tables will be generated by the study statistician and a second statistician from our study team will validate the tables and the code that was used to create them. The tables will then be provided to site pharmacies for packaging.

To ensure adherence to the protocol, viability of the drug delivered and participant safety, at any given time no more than two prepared kits will be available on hand in the ED. The maximum storage time for the kits will be 12 months or until vial expiry. All kits prepared by the pharmacy will be recorded by the pharmacy on the “*Sterile Manufacturing/Packaging Record*” along with the study label. The complete kit will be stored between 15 to 30°C until expiry. Every kit created requires signatures by the pharmacy assistant creating the kit and independent verification by another pharmacy employee. All kits dispensed are recorded in the “*Pharmacy Dispensing Log*” and the log will include participants’ ID and initials, and the total volume of IN interventions dispensed and destroyed according to hospital policy. Even though the pharmacy will maintain this information under their dispensing log, it is also the responsibility of the clinical research coordinator to retain this information using the same or similar accountability log and keep the information in the regulatory binder. This system of double verification ensures that the pharmacy process to create these drug kits has independent oversight to ensure participant safety and protocol adherence.

**6.3 Concomitant Therapy**

For the purposes of this trial, prescription medication is defined as a medication that can be prescribed only by an authorized/licensed clinician. Medications to be reported in the Case Report Form (CRF) are concomitant prescription medications, over-the-counter medications and supplements. The date, time, dose, and nature of all medications (over-the-counter and prescription) administered to the child in the ED and within 24 hours of arrival to the ED will be recorded.

**6.3.1 Co-interventions:**

Based on the judgment of the health care professionals (HCPs), enrolled children may receive any non-pharmacologic or pharmacologic analgesic **but not an anxiolytic or sedative prior to administration of the trial interventions**. In our pilot study of intranasal dexmedetomidine (IND), no additional analgesics or antiemetics were given to any children (47). Pre-intervention anxiolytics or sedatives may be an effect modifier for efficacy outcomes and may compound the anxiolytic effect of the study interventions, placing the child at risk of a severe adverse effect (SAE) (65) However, additional analgesics, anxiolytics, or sedatives (any agent) may be required during the preparation time for laceration repair and may be given based on the judgment of the treating physician to facilitate suture repair. An antiemetic may be given at any time during or following the ED visit.

**6.4 Discontinuation of Study Intervention**

Discontinuation of study interventions includes a decision of the participant, caregiver to not receive or health care provider to not administer the calculated dose of a study intervention once randomized. Participants are free to withdraw from participation in the study at any time upon request. However, data accrued from the participant to the time of withdrawal will be retained by the investigators for analysis. An investigator or treating physician may discontinue or withdraw a participant from the study for the following reasons:

- If any clinical adverse event (AE), or other medical condition or situation occurs such that continued participation in the study would be detrimental to the health of the participant.
- If the participant is found to meet an exclusion criterion (either newly developed or not previously recognized) that precludes further study participation.

The reason for participant discontinuation or withdrawal from the study will be recorded on the Case Report Form (CRF). For the purposes of this trial, a protocol deviation will be defined as any accidental or unintentional change or non-compliance with the Research Ethics Board (REB) approved protocol which increases or decreases benefit, affects the subject’s rights, safety or welfare and the integrity of the study. In the event that a participant is found to meet an exclusion criterion (either newly developed or not previously recognized), this constitutes a protocol deviation and the protocol will be discontinued. However, the participant will be followed for the study period for AEs. A protocol deviation that affects the safety of subjects is regarded as a major protocol deviation and a report must be completed as per local REB requirements and notification should be sent to the local REB and to Western University (Research ethics board) by email. A note to file signed by the site PI should be completed and if any clinical adverse event (AE) occurs, the AE log should also be completed and initialled by the PI. A copy of all documents must be sent to the local REB and to Western University (Research ethics board).

Participants who sign the informed consent form and are randomized but do not receive the study intervention will be analyzed in the groups to which they were randomized as per the ITT principle. The reason for not receiving the intervention will be documented by the research nurse. In addition, participants who sign the informed consent form, are randomized, receive the study intervention, and are subsequently withdrawn will not be replaced by another participant and will be included in the analysis.

**6.5 Rescue medication**

If the participant is not adequately sedated and the laceration repair has begun, the treating physician or their designate may provide any additional analgesics or sedatives they deem necessary for the purposes of facilitating repair. If additional anxiolytic or sedative is given during repair to participants in the N2O group, research nurses or RT will stop administering N2O. Participants who have discontinued the intervention will be analyzed into the group they were originally assigned as per intention to treat (ITT). Acceptance of nasal medication by children is important to inform clinical adoption of the IN interventions. Therefore, the proportion of IN sprays administered and acceptance of face mask will be measured as a secondary outcome. Any medication for management of nausea or vomiting is permitted at any time.

**7.0 Outcomes**

The **primary outcome** is the weighted mean anxiolysis score using the Observational Scale of Behavioral Distress – Revised (OSBD-R) from initial positioning to tying of the last suture. The OSBD-R ranges from 0 (no distress) to 23.5 (maximal distress) (41) and will be scored by two independent outcome assessors remote from the clinical encounter using video capture of the laceration repair due to difficulties scoring the OSBD-R in real-time. Patients who received additional sedation beyond that of the study interventions will be assigned an OSBD-R score of 23.5 (maximal distress). Outcome assessors will code for the presence or absence of 8 child distress behaviors (cry, scream, restraint, verbal resistance, information-seeking, solicitation of emotional support, verbal pain, and flail) during 15-second continuous intervals from initial positioning to tying of the last suture. It is estimated that the duration of the recording will be between 10-15 minutes.

The presence or absence of each behavior are made within each 15-second interval. A *distress score* is then calculated by summing the number of 15-second intervals during which each behavior occurred and multiplying by an expert-determined weight from 1 to 4 based on the severity of the behavior (eg. flailing is weighted more than information seeking). Weightings were prespecified and informed by previous studies (48) and consultation with our patient partner, certified child life specialists (CCLSs), nursing and sedation leads. The will be 4 scoring periods: Phase I (pre-preparation): 3 minutes prior to initial positioning; Phase II (preparation): initial positioning to completion of cleaning, draping, +/- infiltration of additional anesthetic; Phase III (suturing): first suture to tying knot of last suture; Phase IV (recovery): tying last knot to 3 minutes post-procedure. Phases were defined based on the expected window of onset of anxiolysis of all trial interventions so as not to bias the *Total Distress Score* towards a lower value for anxiolytics with longer onsets of action. The weighted scores for each phase are then summed and divided by the number of 15-second intervals, providing a *Total Distress Score*. Given the frequency with which OSBD-R scores will be recorded, we will obtain a video of the child during laceration repair. Videos will be scored by 2 bilingual undergraduate outcome assessors remote from the ED. They will be alerted to the start of each phase using a laminated card. As part of our pilot study (47), 2 independent outcome assessors were trained to score the OSBD-R. This involved a didactic session, followed by concurrent scoring of a video segment of a child undergoing laceration repair with the NPA with open discussion of the meanings of child actions and vocalizations. Outcome assessors then independently scored videos of children undergoing laceration repair until they obtained > 80% interrater agreement (κ). Finally, 2 outcome assessors independently scored videos from all 55 children and the κ during phases I-III were 0.81, 0.87, and 0.88, respectively. Two outcome assessors recruited for the trial will undergo the same training. The OSBD-R has been validated for video capture of children undergoing painful medical procedures and has been used in children undergoing laceration repair (22).

For security purposes, the video will be recorded using a Sony HDR-CX405 HD Handycam or Canon VIXIA HF R700 camcorder (not equipped with WiFi or Bluetooth) and stored on an SD card of 32GB. The camera will be set on a 58’’ tripod with a 3-way pan head to provide stability. Once the video has been recorded, the research nurse will upload the video onto an online platform called Sync^TM^. Sync^TM^ is a Canadian cloud-based service that follows all federal and provincial data privacy guidelines. Sync’s zero-knowledge storage platform guarantees your privacy by providing end-to-end encryption and provides access only to a small group of individuals on the research team. All videos recorded and uploaded onto Sync^TM^ will be deleted when the study is complete. All study data obtained, apart from the video, will be uploaded and stored on the Research Electronic Data Capture (REDCap) platform. This will allow accurate recording of study data and ensure that the data is not accessible to anyone outside the research team.

**7.1 Secondary outcomes**:

- Delayed maladaptive behaviors using the PHBQ (49) < 72 hours collected via telephone or email follow-up from caregivers

- Need for additional anxiolytic or procedural sedation during the procedure Phases II or III

- Adverse effects based on the Quebec guidelines for sedation in children (50)

- Need for physical restraint in Phases II or III using the *Procedural Restraint Intensity in Children* (PRIC) scale (51)

**7.2 Other endpoints:** will be measured by the research nurse during the initial ED visit:

- Compliance (% of calculated IN volume given for IN dexmedetomidine and IN midazolam and willingness to accept nitrous oxide (yes/no)

- Bedside nurse, physician, parent, and child > 7 years satisfaction with anxiolysis using a 100 mm Visual Analog Scale - Nasal irritation during INM and IND administration using *Faces, Legs, Activity, Cry, Consolability (FLACC)* scale (52)

- Caregiver anxiety related to laceration repair using the short version of the State Trait Anxiety Inventory – State (STAI-S) scale (53) will be used because parental anxiety surrounding a medical procedure their child is about to undergo predictors children’s pain and distress during the procedure (53) so may be an effect modifier for OSBD-R scores (54).

- Post-intervention length of stay

- Post-procedure length of stay

- Total ED length of stay

- Duration of procedure

- Maximum number of HCPs required at the bedside during Phase II-IV for restraint, redirection, or care

- Personnel salary costs associated with time at bedside

- Costs to manage AEs

**8.0 Enrollment timeline**

Patients with lacerations will be screened as potential participants.

Assessment of eligibility by inclusion/exclusion criteria; obtain informed consent

**Screening - ED**

**Enrollment/Baseline** **Evaluation** - **ED**

Obtain demographics: age, sex, gender or gender expression (using the GIQC), race and ethnicity (based on CIHI definitions), medical history, weight, size of laceration, location of laceration and vital signs

IN dexmedetomidine and IN midazolam will be administered 30 minutes and 15 minutes prior to laceration repair respectively, and after LET is applied.

**Post-Intervention Assessment**

(Dexmedetomidine) 3 mcg /kg x _____kg / 100mcg/mL =_____ mL

(Midazolam) 0.4mg/kg x_____kg/ 5mg/mL =_______mL

**Dose calculation/ ED**

**Intervention**

**Follow Up: complete PHBQ +/- 72-96 hours**

**Outcome Assessor**

Outcome assessor will score the videos for Observational Scale of Behavioral Distress – Revised (OSBD-R) from Phases 1 – IV

Research nurse records satisfaction from caregiver bedside nurse, health care provider performing repair using a 100 mm VAS

Research nurse will score nasal irritation using the FLACC scale in participants randomized to the IND and INM groups immediately following sprays.

**Nasal Irritation**

Complete all End of study form, Research coordinator records PHBQ Questionnaire by telephone or internet survey

**9.0 Sample Size**

Please refer to Section 15. Statistical Methods

**10. Recruitment**

A research nurse will screen participants during peak volume (evening hours), 5 days a week, 48 weeks a year at each site and record data in the ED using REDCap (55). Children with lacerations will be seen by the ED physician or their designate (any healthcare professional who has the capacity to assess and treat a patient) and determine with the research nurse if the child meets eligibility criteria. If eligible, the research nurse will explain the study protocol and seek informed consent (and assent when appropriate). Prior to enrollment, the research nurse will confirm eligibility using an electronic checklist on REDCap. Written consent will be collected for all potential participants prior to screening. The research nurse will record the demographic features and eligibility criteria of all children who present to the ED with a laceration during the time in which study coverage is available, whether randomized or not, to assess for enrolment bias. This will be entered into a Screening, Eligible, Missed, Other (SEMO) log.

Retention:

It is unlikely that failure of retention will be an issue as the majority of the protocol’s data collection phase and study-related tasks will be completed in the ED and take approximately 1-2 hours. A follow-up phone call to the family will be made between 72 to 96 hours post-discharge to identify maladaptive behaviors related to sedation reflective of the first 24 hours post-sedation. Nevertheless, we want to ensure that participants will remain in the ED long enough post-procedure to collect all secondary outcomes. It will be important to engage caregivers by emphasizing the benefits the study may bring to other children undergoing a similar procedure. Participants will be offered a token of appreciation in the amount of $50 that will be giving to the family as a gift card before they are discharged from the emergency department.

**Methods: Assignment of intervention**

**11. Allocation/ Sequence Generation/ Allocation Concealment Mechanism**

The allocation sequence will be generated using randomize.net. The sequence will be uploaded into REDCap, which will perform randomization in a 1:1:1 ratio using varying block sizes.

**12. Blinding**

Blinding of participants, HCPs, research personnel, and outcome assessors will not be possible due to the nature of the interventions. We considered several approaches to blind outcome assessors including a double-dummy approach where children randomized to IND or intranasal midazolam (INM) received masks delivering room air. However, based on the opinions of our patient partner, CCLS, and clinical expert in N_2_O, we believed this would be an effect modifier for the primary outcome because it may increase distress. We also considered editing videos to pixelate the child’s face and HCP. However, this may invalidate OSBD-R scoring of items such as flailing and restraint. However, several measures will be taken to minimize the risk of ascertainment bias. Prior to involvement in the study, outcome assessors will be recruited from a pool of undergraduates without any clinical experience and screened to ensure they have no preconceived notions about the relative effectiveness of the interventions. They will be told that “this is a trial of 3 anxiolytics for laceration repair” and will be kept unaware of the study objectives and hypotheses. They will also score videos in a remote setting from the ED to avoid being privy to any dialogue about the interventions. HCPs and research personnel will be asked to avoid any commentaries related to the effectiveness of the interventions while recording. Outcome assessors will score all videos independently and an interrater agreement will be generated. Intervention assignment will be concealed from the clinical team until after eligibility has been determined, thereby minimizing the risk of selection bias. Finally, although the OSBD-R is not an objective measure, data analysts will be blinded. The OSBD-R is widely used to measure behavioural distress in paediatric trials, but to date, clinical trials are persistently limited by the inability to blind outcome assessors.

**12.1 Unblinding Procedures**

**Data Safety Monitoring Board (DSMB)**: The DSMB may request unblinding from the statistician holding the master list if they deem it necessary to review possible adverse events (AEs).

**Unblinding:**

**Data Safety Monitoring Board (DSMB)**: The DSMB may request unblinding from the senior statistician holding the master list if they deem necessary to consider the results of the interim safety analysis or to review possible adverse events (AEs).

**Urgent unblinding:**Unblinding in the clinical setting will not be necessary due to the nature of the interventions. However, the DSMB will be blinded and the DSMB may request unblinding from the statistician holding the master list if they deem necessary to review possible adverse events (AEs).

Circumstances that may require urgent unblinding would include concerning clinical symptoms that could be related to the study medications. Unblinding may be performed, in REDCap, only by individually authorized members of the study team. Any unblinding decision may be considered an unanticipated problem and as such will be documented and reported to the DSMB and the institutional REB.

**Methods: Data collection, management and analysis**

**13. Data Collection**

Potential participants will be screened and enrolled consecutively during the hours of research nurse availability. Children with lacerations will be seen by the ED physician or their designate (any doctor who has the capacity to assess, manage, and discharge a patient). The physician or their designate will determine if they need procedural sedation and suture to repair the laceration. The physician will invite the research nurse, if permitted by the family, to screen the child for eligibility. If eligible, the research nurse will explain the study protocol and seek informed consent (and assent when appropriate). Prior to enrollment, the research nurse will confirm eligibility using an electronic checklist on REDCap. All data collection will be completed using REDCap, please find the list of data collection on table 1.

Table 1

| Data collection | |
| --- | --- |
| Demographics | Age |
|  | Sex |
|  | Race and Ethnicity (optional) based on CIHI categories: Black, East Asian, Indigenous (First Nations, Inuk/Inuit, Métis), Latin America, Middle Eastern, South Asian, White and Another race category can be specified. |
|  | Gender (masculine/feminine) or gender expression (-2-12) using parent-reported GIQC if child cannot report gender |
|  | Eligibility criteria |
|  | |
| Injury related | Estimated length of laceration |
|  | Location of laceration |
|  | Number of sutures |
|  | |
| Intervention related | Compliance (volume of drug received/; yes/no tolerance of nose piece) |
|  | Need for additional sedative/anxiolytic/analgesic medication |
|  | Need for physical restraint |
|  | Compliance (volume of drug received/volume of drug prescribed; yes/no tolerance of nose piece) |
|  |  |
|  | |
| Outcomes | OSBD-R (41) |
|  | Nasal irritation using the FLACC scale |
|  | Sedation related adverse events |
|  | State Trait Anxiety Inventory – State (STAI-S) scale (54) |
|  | Delayed maladaptive behaviors < 72 hours post discharge |
|  | |
| ED metrics | Total length of stay |
|  | Post procedure length of stay |
|  | Post intervention length of stay |
|  | Duration of procedure |
|  | Personnel salary costs associated with time at bedside. |
|  | Costs to manage AEs. |
|  | Number of healthcare personnel required at bedside. |
|  | |
| Satisfaction | Caregiver, participant, nurse or respiratory therapist, and procedure physician satisfaction measured using a 100 mm visual analog scale (VAS) |

**14. Data Management**

Data collection and entry is the responsibility of the clinical trial staff at the site under the supervision of the site investigator. The investigator will be responsible for ensuring the accuracy, completeness, legibility, and timeliness of the data reported. All source documents will be completed in a neat, legible manner to ensure accurate interpretation of data. Paper records (eg copies of consent and assent forms) will be stored exclusively in research office in a locked cabinet at each site. A Project Management Plan (PMP) will be developed to provide an overview of the project and provide written structure to carry out a project successfully. It assigns roles and specific tasks throughout. Both DMP and PMP will be developed in accordance with the requirements of the Tri-Council Policy Statement: Ethical Conduct for Research Involving Humans. Data will be entered into a validated electronic, web based, data capture system (REDCap) and will be managed according to approved DMP. During the data collection process data may be collected on paper and transcribed into the study database or, in some cases, information obtained directly from the participants may be entered directly into the study database. Under these circumstances the study database may be considered to be an electronic source document. Selected data elements will be validated electronically on an ongoing basis throughout the study and any discrepancies will be assigned to members of the study team for resolution.

The study team will provide hardcopies of the study worksheets for use as source document for recording data for each participant enrolled in the study if REDCap or Internet access is unavailable. Hardcopy data will be entered into REDCap as soon as the online systems are available. Data derived from hardcopies should be consistent with the data recorded on the source documents.

Clinical data (including adverse events (AEs), concomitant medications, and expected adverse reactions data) and clinical laboratory data will be entered into REDCap. REDCap includes password protection and internal quality checks, such as automatic range checks, to identify data that appear inconsistent, incomplete, or inaccurate. Clinical data will be entered directly from the source documents, participants, or study team.

The site investigator will be responsible for retaining (archiving) their own essential study documents that individually or collectively permit the evaluation and conduct of the study and the quality of data, in accordance with International Conference on Harmonisation – Good Clinical Practice (ICH-GCP) and applicable regulatory requirements. All study documents, including source, are to be stored in a confidential location with secured and limited access. All electronic records and data sets will be encrypted and password protected with access only permitted by the PI, site coordinator(s), and research team members. Paper data (e.g. copies of consent and assent forms) will be stored exclusively in the Participating Site Investigator’s research office in a locked cabinet. Results will not be reported in a way that identifies any individuals.

All study related documentation will be retained in accordance with Health Canada’s Food and Drug Regulations for 15 years and per the investigational site’s institutional record management and retention policies. No records will be destroyed without the written consent of the Qualified Investigator and/or Sponsor.

**15. Statistical Methods**

We will use a novel Bayesian adaptive method to ensure timely completion of the recruitment period, reduce the operational costs of conducting the trial, and the number of children are unnecessarily randomized (39). Based on interim analyses determined *a priori*, a trial arm with poor efficacy, intolerability, or undesirable adverse effects such as prolonged sedation, will be dropped pending a single interim analysis for futility. Starting with N=159 (53 per arm), the interim analysis will proceed as follows:

1. If two treatments are considered futile based on our pre-specified dropping rule, we will designate that the remaining treatment is superior, and the trial sample size will remain at 159 (53 per arm), accounting for 5% dropouts, since no more participants are required.
2. If one treatment is considered futile based on our pre-specified dropping rule, the total sample size will be 263, accounting for 5% dropouts.
3. If no treatment is considered futility based on our pre-specified dropping rule, the total sample size will be 315, accounting for 5% dropouts.

Our pre-specified dropping rule and final analysis will be based on the probability that a given intervention ranks high on efficacy (P*_best_*).(46) At the interim analysis, a treatment will be considered futile if P*_best_* is < 0.026, at the final analysis, a treatment will be considered superior if P*_best_* is > 0.975. Simulations have been undertaken to ensure that the ALICE trial has type I error rate < 2.5%. The power of the ALICE trial is 82% and 85% for an MCID of 1.0 and 1.5, respectively. The overall power of the trial is 80%.

Statistical analysis plan

| **Research Question** | **Outcome Measure** | **Statistical Model** | **Reporting** |
| --- | --- | --- | --- |
| **Primary Outcome** | | | |
| What is the optimal anxiolytic for laceration repair in children? | OSBD-R *Total Distress Score* | The mean *Total Distress Score*^1^ and pain will be estimated using a Bayesian linear model, adjusted for site, age, type of proceduralist (staff physician, trainee), pre-intervention *Total Distress Score,* pre-intervention analgesia, parental anxiety., sex, gender, and gender-expression. For *Total Distress Score*, we will conduct a subgroup analysis using a Bayesian linear model with a test of interaction based on sex (male/female), gender (masculine/feminine), and gender-expression (cross- versus same-gendered). | We will report posterior means, standard deviations (SD), and 95% high density posterior credible intervals for the adjusted mean effectiveness for each anxiolytic. |
| **Secondary Outcomes** | | | |
| Which anxiolytic is associated with the fewest late maladaptive behaviors? | Late maladaptive behaviors using the *Post-Hospital Behavior Questionnaire* *(PHBQ)* | Descriptive statistics for the *PHBQ* | For each anxiolytic, we report five-number summary, mean, SD and plots for the *PHBQ*. The optimal anxiolytic (with the fewest late maladaptive behaviors) will be recommended using the descriptive statistics. |
| Which anxiolytic is associated with the fewest participants requiring additional anxiolysis or sedation | Need for additional anxiolytic or procedural sedation | Bayesian generalized linear model with Bernoulli distribution (additional drug vs. no additional drug), and the logit link, adjusted for site. | We will summarize the posterior distribution of the proportion of additional drug for each anxiolytic or sedative using posterior means, standard deviations (SD), 95% high density posterior credible intervals. We will also report the proportion with significant behavioral changes, defined as > 7 negative behaviors. |
| Which anxiolytic is associated with the fewest participants requiring physical restraint? | Need for physical restraint using the *Procedural Restraint Intensity in Children* (PRIC) scale | Bayesian generalized linear model with Bernoulli distribution (physical restraint vs. no physical restraint), and the logit link, adjusted for site. | The posterior distribution of the proportion of physical restraint for each anxiolytic. Posterior means, standard deviations (SD), 95% high density posterior credible intervals., and plots are reported. |
| Which anxiolytic is associated with the most frequent adverse events (AEs)? | AEs | Bayesian generalized linear model with Bernoulli distribution (AE vs. No AE) and the logit link for each AE, adjusted for site. | We will report severity, frequency, duration, and relationship of AEs to the intervention according to the *MeDRA*^2^. We will also report the severity, frequency, and relationship of AEs to biological sex. For each AE, we will report the start date, stop date, severity, relationship, expectedness, outcome, and duration. AEs leading to premature discontinuation from the study intervention and SAEs will be presented in a table. We will also summarize the posterior distribution of the proportion of AEs for each anxiolytic using posterior means, standard deviations (SD), 95% high density posterior credible intervals. |
| **Additional Endpoints** | | | |
| Which anxiolytic is associated with the greatest compliance with intervention administration? | Compliance with intervention administration (INM and IND: % of calculated volume administered; N_2_O: willingness to accept mask or mouthpiece to achieve desired level of anxiolysis (as per physician’s judgment (yes/no)) | Descriptive statistics for the proportion of compliance. | For each anxiolytic, we report sample size, proportions, the number of patients experiencing compliance and side-by-side bar plots. The optimal anxiolytic (with the greatest compliance) will be recommended using the descriptive statistics. |
| Which anxiolytic is associated with the highest satisfaction? | Bedside nurse, respiratory therapist (if any), physician, caregiver, and child (> 7 years) satisfaction with anxiolysis using a 100 mm Visual Analog | Descriptive statistics of satisfaction. | For each anxiolytic and Likert scale, we report the number of patients, median, proportions and bar plots. The optimal anxiolytic (highest satisfaction) will be recommended using the descriptive statistics. |
| Which intranasal anxiolytic is associated with the least nasal irritation during administration | Nasal irritation during IND and INM administration using *Faces, Legs, Cry, Consolability (FLACC)* scale | Descriptive statistics for the *FLACC* scale. | For each anxiolytic, we report five-number summary, mean, SD and plots for the *FLACC* scale. The optimal anxiolytic (with the lowest score) will be recommended using the descriptive statistics. |
| Which anxiolytic is associated with the shortest duration of procedure, post-procedure and total length of stay (LOS) | Duration of procedure (initial positioning to tying of knot of last suture), post-procedure LOS (tying of knot of final suture to discharge) and total LOS (triage to discharge time according to medical record) | Descriptive statistics for LOS | For each anxiolytic, we report five-number summary (minimum, 1^st^ quantile, median, 3^rd^ quantile, maximum), mean, SD and plots for these parameters. The optimal anxiolytic (with shortest post-procedure and total LOS) will be recommended using the descriptive statistics. |
| Which anxiolytic is the most cost-effective? | ED LOS; salary costs associated with HCP and research personnel at the bedside; drug costs; costs associated with managing AEs | Bayesian individual-level phase-transition model, incorporating prior information of adequate sedation, rescue anxiolytic or procedural sedation, costs of managing AEs, ED LOS, drug and personnel costs. | We will summarize the total expected ED LOS, the expected costs for each anxiolytic or sedative using means, standard deviations (SD), 95% high density posterior credible intervals, and density plots. We will calculate the probability that each intervention minimized the cost and perform sensitivity analyses by calculating the expected value of perfect information (EVPI) and expected value of partial perfect information (EVPPI). |
| Which anxiolytic is associated with lowest caregiver anxiety related to laceration repair? | State anxiety using the caregiver-reported *State Trait Anxiety Short – 5 Item* (STAIS-5) | Descriptive statistics | For each anxiolytic, we report the mean, SD and proportion meeting threshold (score ≥10) reflecting potentially clinically anxious. |
| What is the degree of clinical adoption of trial findings? | Proportion of children undergoing laceration repair that receive anxiolysis based on trial findings | Descriptive statistics | We will report demographic variables and structure, process, and outcome measures outlined in Appendix E using means and standard deviations, medians and interquartile ranges, or percentages, as appropriate. The frequency of identified causes from the root cause analysis will be displayed using a Pareto Plot. Before and after comparisons of the outcome measure will be conducted using a statistical process control analysis. |

**Missing data:** The intention to treat principle will be used. We expect minimal missing data as the primary outcome is collected during laceration repair before discharge. Thus, sample size will not be adjusted for loss to follow-up. If the proportion of missing data is < 5%, we will undertake a complete case analysis. If the missingness > 5%, we will use a joint Bayesian model for the missingness and outcome.

For *Total Distress Score*, we will conduct subgroup analyses using a Bayesian linear model with a test of interaction between group and the following: i) sex because of differential responses to analgesia (56); ii) gender (masculine/feminine) and gender-expression (cross- versus same-gendered) because of potential differences in expression of distress (57, 58). These will be exploratory analyses the sample size will not be adjusted. Bayesian hierarchical models will adjust *Total Distress Score* for potential risk modifiers: site; age; staff physician versus trainee; pre-procedural *Total Distress Score;* pre-procedural analgesia; parental anxiety.

We will also conduct an economic analysis. The primary cost-effectiveness analysis will follow *Canadian Agency for Drugs & Technologies in Health Guidelines* on the conduct of economic evaluations in Canada (59, 60). Costs will be adjusted for inflation using the Canadian Consumer Price Index and reported in 2025 Canadian dollars. We will take a health system cost perspective, with all costs associated with the interventions accounted for including Total Distress Score, ED length of stay, medication costs, ED personnel costs for time spent at the bedside, and the costs of managing AEs. We will use an individual-level state transition model to evaluate the total health-service costs up to 72 hours post-discharge. Effectiveness will be measured using the OSBD-R as Quality Adjusted Life Years (QALYs) are less relevant in children (61) and unable to evaluate the effectiveness of short-term interventions. We will undertake a full probabilistic analysis by analyzing the data from the ALICE trial under a Bayesian framework (62). We will report the incremental cost-effectiveness ratio (ICER) comparing total costs to Total Distress Score(63) and the mean, standard deviation, 95% interval for the OSBD-R, hospital length of stay and total costs. Results will be reported according to the Consolidated Health Economic Evaluation Reporting Standards 2022 (64).

**Methods: Monitoring**

**16. Data Monitoring**

Safety oversight will be under the direction of a Data and Safety Monitoring Board (DSMB) composed of individuals with the appropriate expertise, including trial methodology, epidemiology, pharmacology, biostatistics and pediatric emergency medicine. Members of the DSMB should be independent from the study conduct and free of conflict of interest, or measures should be in place to minimize perceived conflict of interest. The DSMB will meet biannually or ad hoc to assess safety and efficacy data on each arm of the study.

The DMSB will operate under the rules of an approved charter/terms of reference that will be reviewed at the organizational meeting of the DSMB. At this time, each data element that the DSMB needs to assess will be defined. The DSMB will provide its input to the PI, Qualified Investigators, and Steering Committee. The DSMB may also provide input to the responsible REB, and Health Canada as requested.

Interim analyses will be done once 53 participants per arm have been enrolled. As described in the statistical analysis, the interim analyses determined *a priori*, a trial arm with poor efficacy, intolerability, or undesirable adverse effects such as prolonged sedation, will be dropped.

The DSMB will, in collaboration with the trial research team will revise the stopping rule in place and establish safety stopping rules prior to trial initiation.

Stopping rules: circumstances that may warrant termination or suspension include, but are not limited to:

- Determination of unexpected, significant, or unacceptable risk to participants
- Demonstration of efficacy that would warrant stopping
- Insufficient compliance with protocol requirements
- Data that are not sufficiently complete and evaluable
- Determination that the primary endpoint has been met
- Determination of futility

This study may be temporarily suspended or prematurely terminated if there is sufficient reasonable cause based on the findings of the DSMB of futility or safety issues. The latter include determination by the DSMB of unexpected, significant, or unacceptable risks to participants such as complications due to treatment or related adverse events at rates above expected or where it can be demonstrated that efficacy warrants stopping the study early. Based on the DSMB Charter, the DSMB will make recommendation to the Steering Committee. Written notification, documenting the reason for study suspension or termination, will be provided by the suspending or terminating party to study participants, Participating Site Investigators, funding agency, the Sponsor, responsible REB and Health Canada. If the study is prematurely terminated or suspended, the Qualified Investigator will promptly inform Participating Site Investigators. The Participating Site Investigators will then inform study participants, their responsible REB. The Qualified Principal Investigator will provide the reason(s) for the termination or suspension. Study participants will be contacted, as applicable, and be informed of changes to study visit schedule. The study may resume once concerns about safety, protocol compliance, and data quality are addressed, and satisfy the Sponsor, REB and/or Health Canada.

**16.1 Harms**

Maladaptive behaviors due to sedation: This will be assessed by the research nurse or research coordinator using the Post-Hospital Behavior Questionnaire (PBHQ) administered by phone or email survey 72 to 96 hours (+ 14/days window) following discharge to screen for delayed maladaptive behaviors and adverse events within 72 hours of discharge. This information will be recorded using REDCap (Dose Finding Study PHBQ form). The extra 14-day window for survey completion provides flexibility for participants who may face challenges finishing the survey within the initial 72 to 96-hour timeframe. Surveys completed within this extended period must reflect responses corresponding to the required 72 to 96 hours after discharge.

**Follow-Up Procedure for Discharge Data Collection**

1. **Automated Email Surveys:**
   - **Timing:** REDCap will automatically send follow-up emails to caregivers who prefer email surveys 72-96 hours after discharge.
   - **Reminders:** If the email survey is not completed, REDCap will send up to two reminder emails, spaced 7 days apart.
2. **Telephone Surveys:**
   - **Initial Call:** For caregivers who prefer a telephone survey, the first call will occur 72-96 hours post-discharge.
   - **Subsequent Attempts:** If the caregiver is unreachable, a minimum of three contact attempts must be made, with attempts spaced at least 7 days apart.
   - **Weekends and Holidays:** If the follow-up period includes a weekend or holiday, and the initial call is delayed, ensure that a minimum of three attempts are made once the research nurse or coordinator is available.
3. **In-between Contact Attempts:**
   - If a survey is missed, or reminders have been sent by REDCap but the survey is still incomplete, the research nurse or coordinator will follow up via telephone between REDCap’s automatic reminders.
4. **Data Collection Compliance:**
   - Ensure that all caregivers provide their follow-up data considering the 72-96 hour window after discharge, even if completing a late PHBQ survey.

Nasal irritation: The research nurse will rate pain for participants in the IND and INM groups immediately following nasal sprays using the FLACC scale.

Vital Signs: Heart rate (HR), systolic blood pressure (SBP), diastolic blood pressure (DBP), oxygen saturation (SpO2), and respiratory rate (RR) will be recorded at baseline (by the triage nurse at the time of triage). Monitoring of these vital signs following administration of the intervention will be carried out by the research nurse and/or the bedside nurse. The research nurse will monitor vital signs every 5 mins post-intervention until the participant is deemed ready for discharge based on site-specific policies. The research nurse will record the vital signs in the participant source folder and enter them into REDCap. The bedside nurse will monitor patients as per site specific policies.

If the participant’s has a significant change in vital signs such as SpO2 decrease to < 90%, Systolic blood pressure decrease below the threshold SBP (a systolic pressure drop > 30% from baseline), HR decrease to < 60 beats per minute, respiratory paused > 10 seconds AND requires any one of the following intervention: chest compressions, positive pressure ventilation, endotracheal intubation, oral airway, jaw thrust, intravenous fluid bolus for the purposes of correcting abnormal vital signs, or inotropic support, the type of intervention used and the starting time the vital sign change as well as the time the vital sign returned to normal will be collected and entered to REDCap (Vital signs CRF forms).

Length of stay (LOS): Defined as the time interval between triage assessment and discharge.

Post-procedure (PPLOS): Defined as the time interval between tying of the last suture to discharge.

Duration of procedure: Defined as time interval between initial position to tying of the last suture.

Length of stay due to intervention: Defined as time interval from intervention administration to discharge.

Adverse events (AEs): The research nurse will be trained on the recognition and definition of all expected and unexpected AEs. AEs are document medical events that occur to a participant/subject once enrolled in a study. AEs are the construct through which the safety of an intervention is recorded and assessed during the study period. All AEs will be collected using REDCap. The form will include the definitions and AE descriptions that could be related to sedation. Uncertainty regarding the presence of AEs will be clarified with the sedating physician (if it occurs while participant is in the paediatric emergency department) and with PI for all AE cases reported.

**16.2 Reporting AEs, Serious Adverse Events and Unexpected Drug Reactions**

**All adverse events (AEs)** will be reported to the Research Ethics Board in accordance with site’s AE reporting guidelines. The PI will assess each AE in terms of its expectedness and relationship to the study drug. Information to be collected will include an event description, date of onset, clinician’s assessment of severity, relationship to study intervention (assessed only by those with the training and authority to make a diagnosis), and date of resolution/stabilization of the event and event outcome (resolved/recovered, recovered with sequalae, not recovered/not resolved, death, or unknown).

The following guidelines will be used to describe grade severity:

- **Mild** – Events require minimal or no treatment and do not interfere with the participant’s daily activities.
- **Moderate** – Events result in a low level of inconvenience or concern with the therapeutic measures. Moderate events may cause some interference with functioning.
- **Severe** – Events interrupt a participant’s usual daily activity and may require systemic drug therapy or other treatment. Severe events are usually potentially life-threatening or incapacitating. Of note, the term “severe” does not necessarily equate to “serious”.

Relationship with study intervention:

All adverse events (AEs) must have their relationship to study intervention assessed by the clinician who examines and evaluates the participant based on temporal relationship and his/her clinical judgment. The degree of certainty about causality will be graded using the categories below. In a clinical trial, the study product must always be suspect.

- **Definitely Related** – There is clear evidence to suggest a causal relationship, and other possible contributing factors can be ruled out. The clinical event, including an abnormal laboratory test result, occurs in a plausible time relationship to study intervention administration and cannot be explained by concurrent disease or other drugs or chemicals. The response to withdrawal of the study intervention (de-challenge) should be clinically plausible. The event must be pharmacologically or phenomenologically definitive, with the use of a satisfactory re-challenge procedure if necessary.
- **Probably Related** – There is evidence to suggest a causal relationship, and the influence of other factors is unlikely. The clinical event, including an abnormal laboratory test result, occurs within a reasonable time after administration of the study intervention, is unlikely to be attributed to concurrent disease or other drugs or chemicals, and follows a clinically reasonable response on withdrawal (de-challenge). Re-challenge information is not required to fulfill this definition.
- **Potentially Related** – There is some evidence to suggest a causal relationship (e.g., the event occurred within a reasonable time after administration of the trial medication). However, other factors may have contributed to the event (e.g., the participant’s clinical condition, other concomitant events). Although an AE may rate only as “possibly related” soon after discovery, it can be flagged as requiring more information and later be upgraded to “probably related” or “definitely related”, as appropriate.
- **Unlikely to be related** – A clinical event, including an abnormal laboratory test result, whose temporal relationship to study intervention administration makes a causal relationship improbable (e.g., the event did not occur within a reasonable time after administration of the study intervention) and in which other drugs or chemicals or underlying disease provides plausible explanations (e.g., the participant’s clinical condition, other concomitant treatments).
- **Not Related** – The AE is completely independent of study intervention administration, and evidence exists that the event is definitely related to another etiology. There must be an alternative, definitive etiology documented by the clinician.

**A** **Serious Adverse Event (SAE)** will be defined as: any adverse occurrence of a clinical trial subject who is administered a drug at any dose, or placebo that may or may not be caused by the administration of the drug or placebo that results in:

- Hospitalization due to a sedation related event
- Prolongation of existing hospitalization
- Congenital malformation or birth defect
- Persistent or significant disability or incapacity
- An outcome that is life-threatening
- Death

Important medical events that may not result in death, be life-threatening, substantially disrupt one’s ability to conduct normal life functions or require hospitalization may be considered serious when, based upon appropriate medical judgment, they may jeopardize the participant and may require medical or surgical intervention to prevent one of the outcomes listed in this definition.

**SAEs** will be defined based on the Quebec guidelines (<https://www.ncbi.nlm.nih.gov/pubmed/19026467)> and include apnea, laryngospasm, hypotension, bradycardia, airway obstruction, clinically-apparent pulmonary aspiration, permanent neurologic injury or death, or significant interventions performed in response to an SAE (positive pressure ventilation, endotracheal intubation, vasoactive medications or chest compressions) (65) in all groups. The data safety monitoring board (DSMB) will use these recommendations to identify suspected SAEs. Expected and unexpected SAEs will be reported to the DSMB and Health Canada, respectively. A list of unexpected SAEs and definitions can be found on **Appendix IV**

**All serious, unexpected AEs** and drug reactions will be reported to Health Canada by the Qualified Principal Investigator within 15 calendar days after the Qualified Principal Investigator becomes aware of the event. For death or life-threatening events, this report must be done within 7 calendar days after the Qualified Principal Investigator becomes aware of the event. In the latter case, a follow-up report must be filed within 8 calendar days. All AEs will also be submitted, in accordance with the DSMB safety monitoring plan to the independent DSMB assigned to this study.

**All serious adverse events (SAEs)** will be followed until satisfactory resolution or until the PI deems the event to be chronic or the participant is stable. The Qualified Principal Investigator will also, within 8 days after having informed Health Canada of the adverse drug reaction, submit as complete as possible, a report which includes an assessment of the importance and implication of any findings.

**16.3 Monitoring and Auditing**

Clinical site monitoring will be conducted to ensure that the rights and well-being of trial participants are protected, that the reported trial data are accurate, complete, and verifiable, and that the conduct of the trial is in compliance with the currently approved protocol/amendment(s), with International Conference on Harmonization-Good Clinical Practice (ICH-GCP), Tri-Council Policy Statement 2 (TCPS2) and with applicable regulatory requirement(s), such as Health Canada.

Monitoring of data integrity, regulatory compliance and participant safety will be performed in accordance with a Monitoring Plan and will include centralized review of CRFs and other study documents for compliance, data accuracy and completeness. Monitoring may include monitoring visits to investigational sites during the study for source data verification, review of the investigator’s site file and drug handling records. The assigned monitor will be given direct access to source documents, CRFs and other study-related documents by the Participating Site Investigator. By signing the informed consent form, the parent or guardian gives authorized assigned monitor to directly access to the participant’s medical records and the study data. This study may be subject to audit or inspection by representatives of the organization contracted for monitoring or representatives of Health Canada.

**Ethics and Dissemination**

**17. Research ethics approval**

The protocol, informed consent form(s), recruitment materials, and all participant materials will be submitted to the REB and Health Canada for review and approval. Approval of both the protocol and the consent forms must be obtained before any participant is enrolled.

**18. Protocol amendments**

Any amendment to the protocol will require review and approval by the REB before the changes are implemented to the study as well as authorization form Health Canada. All changes to the consent form will be REB approved; a determination will be made regarding whether a new consent needs to be obtained from participants who provided consent, using a previously approved consent form.

**19. Consent or assent**

All caregivers of potential participants will be offered a letter of information (LOI) and consent that will outline the study’s purpose and expectations of the participant. This will be done by research personnel (research nurse, research assistant, bedside nurse, or site research coordinator). This information will provide sufficient information to enable the family to make an informed decision about their participation in the study. The Participating Site’s Research nurse is responsible for answering questions posed to them by the family. The LOI and Consent, and Assent will require prior REB approval before being used in the study. The Consent and Assent must be signed before a participant is submitted to any study procedure. Signed Consent and Assent forms are a condition of participation in the study. The Consent may be signed by the participant or legally acceptable surrogate, and the Participating Site’s Research nurse obtaining the consent. Assent will be sought from potential participants when appropriate. The LOI and Consent forms describing in detail the study intervention, study procedures, and risks are written at a grade 8 level. The Assent form is written at a grade 3 reading level and can be read to the child by the caregiver.

Informed consent/assent is a process that is initiated before the participant and their caregiver agree to participate in the study and continues throughout the individual’s participation. Prior to enrollment, the participant and their caregiver will be asked to read and review the LOI and Assent documents. The research personnel will explain the study to the participant and answer any questions that may arise. A verbal explanation will be provided in terms suited to the participant’s and their caregivers’ comprehension of the purposes, procedures, and potential risks of the study and their rights as research participants. Participants and their caregivers will have the opportunity to carefully review the written consent form and ask questions before signing. The participants and their caregivers will have the opportunity to discuss the study with their family or surrogates or think about it before agreeing to participate. The caregiver will sign the Consent and (if applicable) the participant will sign the Assent document prior to any study-related procedures or data collection. Participants and their caregivers will be informed that participation is voluntary and that they may withdraw from the study at any time, without affecting the quality or timeliness of their care. A copy of the LOI, signed Consent and Assent (if applicable) will be given to the family for their records. The informed consent process will be conducted and documented on REDCap (including the date) before the participant undergoes any study-related procedures or data collection. The rights and welfare of the participants will be protected by emphasizing to them that the quality of their medical care will not be adversely affected if they decline to participate in this study.

Consent will also be sought from the bedside nurse and sedating physician as they will be asked questions pertaining to the sedation and their satisfaction. These will be obtained by the Participating Site’s Research nurse/assistant the start of the trial from all the bedside nurses and sedating physicians and pertain to all participants to be enrolled in the study.

**20. Confidentiality**

Participant confidentiality and privacy are strictly held in trust by the participating investigators, their staff, and the sponsor(s) and their interventions. This confidentiality is extended to cover to the clinical information relating to participants. Therefore, the study protocol, documentation, data, and all other information generated will be held in strict confidence. No information concerning the study or the data will be released to any unauthorized third party without the prior written approval of the sponsor. All research activities will be conducted in as private a setting as possible.

The study monitor, other authorized representatives of the sponsor, representatives of the Research Ethics Board (REB), or regulatory agencies may inspect all documents and records required to be maintained by the investigator, including but not limited to, medical records (office, clinic, or hospital) and pharmacy records for the participants in this study. The clinical study site will permit access to such records. The study participant’s contact information will be securely stored at each clinical site for internal use during the study. At the end of the study, all records will continue to be kept in a secure location for as long a period as dictated by the reviewing REB, institutional policies, or sponsor requirements. For Health Canada regulated trials, this is 15 years. De-identified study participant research data, which is for purposes of statistical analysis and scientific reporting, will be stored in the REDCap electronic data capture system at Lawson Health Research Institute (Lawson). This data will not include the participant’s contact or identifying information. Rather, individual participants and their research data will be identified by a unique study identification number. Permission to store data at the Lawson REDCap will be included in the informed consent.

1. **Declaration of interests**

No member of the research team has any financial or competing interests relevant to the conduct of the study, or the interpretation or dissemination of the results.

1. **Access to data**

All electronic records and data sets will be encrypted and password protected with access only permitted by the PI, site coordinator(s), and research team members. The investigational site will provide direct access to all trial-related source data/documents, and reports for the purpose of monitoring and auditing by the sponsor, and inspection by local and regulatory authorities.

Data will remain in REDCap system at Lawson until all data management and statistical analysis activity has been completed. Following study completion and publication the data will be deleted from the REDCap system. Lawson will facilitate the deposit of de-identified data in a publicly accessible, secure and curated repository for discovery and reuse by others in accordance with the Tri Agency Statement of Principles on Digital Data Management (2021).

The PI will be responsible for storing copies of the data and other study materials in a secure archival facility in compliance with Health Canada and local institutional research data retention policy. At the end of this retention period these materials will be destroyed.

1. **Ancillary and post-trial care**

For any participant who contacted our research team up to and including the 24-48hour follow up period, and the research team perceived that the participant suffered any harm as a result of participation in the trial, the information will be provided to REB and the necessary documentation will be put in place to support or compensate the patient for their needs. The question: do you believe you have suffered any harm due to participation in this study? The response options will be YES or NO.

1. **Dissemination policy**

We will disseminate our findings to EDs beyond the participating sites. Our strategy will be supported by Western’s Knowledge Exchange & Impact Program and guided by the *Solutions for Kids in Pain* template used at Toronto’s Hospital for Sick Children (66). Locally, we will engage our nurse educator, child life specialist, and community ED leads to create a practice guideline for anxiolysis for laceration repair based on our trial results. We will disseminate this to knowledge users using online toolkits, infographics, and in-service workshops to facilitate implementation. Nationally, we will leverage existing relationships with: Skeptics Guide to Emergency Medicine (67) and EMRap to develop podcasts, Children’s Healthcare Canada to host national webinars (68), Translating Emergency Knowledge for Kids to develop a Bottom Line Recommendation (BLR) (69), and Pediatric Emergency Research Canada (PERC) to update the Canadian Paediatric Society’s position statement on procedural distress to include laceration repair (10). We will use Western Media Relations and Scholarship@Western to promote and provide open-access to our materials. We will publish and present in high-impact journals, national conferences, and professional development seminars.

1. **References**

1. O’Donnell M, Williams J. Children’s Emergency Department Services: Setting the Stage for Tiers Development Vancouver, BC.2016 [Available from: <https://www.childhealthbc.ca/sites/default/files/20-07-01-ed-tiers-setting-the-stage-1819-data.pdf>.

2. Hall JE, Dhruv P, Thomas JW, Richards CA, Rogers PE, Pruitt CM. Certified Child Life Specialists lessen emotional distress of children undergoing laceration repair in the emergency department. Pediatr Emerg Care. 2018;34(9):603-6.

3. Kumar K, Ali S, Sabhaney V, Trottier E, Drendel A, Bhatt M, et al. Anxiolysis for laceration repair in children: a survey of pediatric emergency providers in Canada. CJEM. 2022;24(1):75-83.

4. Gursky B, Kestler LP, Lewis M. Psychosocial intervention on procedure-related distress in children being treated for laceration repair. J Dev Behav Pediatr. 2010;31:217-22.

5. Sinha M, Christopher NC, Fenn R, Reeves L. Evaluation of nonpharmacologic methods of pain and anxiety management for laceration repair in the pediatric emergency department. Pediatrics. 2006;117(4):1162-8.

6. Miller JL, Capino AC, Thomas A, Couloures K, Johnson PN. Sedation and analgesia using medications delivered via the extravascular route in children undergoing laceration repair. J Pediatr Pharmacol Ther. 2018;23:72-83.

7. Trottier ED, Ali S, Le May S, Gravel J. Treating and reducing anxiety and pain in the pediatric emergency department: The TRAPPED survey. Paediatr Child Health. 2015;20(5):239-44.

8. Taddio A, McGrath P, Finley A. Effects of early pain experience: The human literature. Progress in Pain Research and Management. 1999;13:57-74.

9. Fein JA, Zempsky WT, Cravero JP, Committee on Pediatric Emergency Medicine and Section on Anesthesiology and Pain Medicine. Relief of pain and anxiety in pediatric patients in emergency medical systems. Pediatrics. 2012;130(5):e1391-405.

10. Doyon-Trottier E, Doré-Bergeron MJ, Chauvin-Kimoff L, Baerg K, Ali S. Managing pain and distress in children undergoing brief diagnostic and therapeutic procedures. Paediatr Child Health. 2019;24(8):509-21.

11. Coté CJ, Wilson S, American Academy of Pediatrics, American Academy of Pediatric Dentistry. Guidelines for monitoring and management of pediatric patients before, during, and after sedation for diagnostic and therapeutic procedures: update 2016. Pediatrics. 2016;138(1):e1-e31.

12. Conway A, Rolley J, Sutherland JR. Midazolam for sedation before procedures. Cochrane Database Syst Rev. 2016;5:CD009491.

13. National Clinical Guideline Centre (UK). NICE Clinical Guidelines No. 112, Sedation in Children and Young People: Sedation for Diagnostic and Therapeutic Procedures in Children and Young People. London: Royal College of Physicians (UK). 2010.

14. ter Bruggen FFJA, Eralp I, Jansen CK, Stronks DL, Huygen FJPM. Efficacy of dexmedetomidine as a sole sedative agent in small diagnostic and therapeutic procedures: a systematic review. Pain Pract. 2017;17(6):829-40.

15. Poonai N, Spohn J, Vandermeer B, Ali S, Bhatt M, Hendrikx S, et al. Intranasal dexmedetomidine for anxiety-provoking procedures in children: a systematic review and meta-analysis. Pediatrics. 2020;145(1):e20191623.

16. Neville DNW, Hayes KR, Ivan Y, McDowell ER, Pitetti RD. Double-blind randomized controlled trial of intranasal dexmedetomidine versus intranasal midazolam as anxiolysis prior to pediatric laceration repair in the emergency department. Acad Emerg Med. 2016;23:910-7.

17. Poonai N, Sabhaney V, Ali S, Trottier E, Heath A. Intranasal dexmedetomidine for laceration repair in children: a dose-finding study using the continual reassessment method. In Preparation. 2021.

18. Tug A, Hanci A, Turk HS, Aybey F, Isil CT, Sayin P, et al. Comparison of two different intranasal doses of dexmedetomidine in children for magnetic

resonance imaging sedation. Pediatr Drugs. 2015;17:479-85.

19. Klein EJ, Brown JC, Kobayashi A, Osincup D, Seidel K. A randomized clinical trial comparing oral, aerosolized intranasal, and aerosolized buccal midazolam. Ann Emerg Med. 2011;58:323-29.

20. Everitt IJ, Barnett P. Comparison of two benzodiazepines used for sedation of children undergoing suturing of a laceration in an emergency department. Pediatr Emer Care. 2002;18(2):72-4.

21. Fantacci C, Fabrizio GC, Ferrara P, Franceschi F, Chiaretti A. Intranasal drug administration for procedural sedation in children admitted to pediatric emergency room. Eur Rev Med Pharmacol Sci. 2018;22:217-22.

22. Luhmann JD, Kennedy RM, Porter FL, Miller JP, Jaffe DM. A randomized clinical trial of continuous-flow nitrous oxide and midazolam for sedation of young children during laceration repair. Ann Emerg Med. 2001;37(1):20-7.

23. Ryan PM, Kienstra AJ, Cosgrove P, Vezzetti R, Wilkinson M. Safety and effectiveness of intranasal midazolam and fentanyl used in combination in the pediatric emergency department. Am J Emerg Med. 2019;37:237-40.

24. Pedersen RS, Bayat A, Steen NP, Jacobsson M-L B. Nitrous oxide provides safe and effective analgesia for minor paediatric procedures – a systematic review. Dan Med J. 2013;60(6):A4627.

25. Hartling L, Milne A, Foisy M, Lang ES, Sinclair D, Klassen TP, et al. What works and what's safe in pediatric emergency procedural sedation: an overview of reviews. Acad Emerg Med. 2016;23(5):519-30.

26. Royal Children’s Hospital Melbourne. Clinical Practice Guidelines Nitrous Oxide - Oxygen Mix 2021 [Available from: <https://www.rch.org.au/clinicalguide/guideline_index/Nitrous_Oxide_Oxygen_Mix/>.

27. Dentistry AAoP. Guideline on Use of Nitrous Oxide for Pediatric Dental Patients. Clinical Practice Guidelines. 2013;37(6):206-10.

28. Brown SM, Sneyd JR. Nitrous oxide in modern anaesthetic practice. BJA Education. 2016;16(3):87-91.

29. Poonai N, Creene C, Dobrowlanski A, Geda R, Hartling L, Ali S, et al. Inhaled nitrous oxide for distressing procedures in children: a systematic review. In Preparation. 2021.

30. Bar-Meir E, Zaslansky R, Regev E, Keidan I, Orenstein A, Winkler E. Nitrous oxide administered by the plastic surgeon for repair of facial lacerations in children in the emergency room. Plast Reconstr Surg. 2006;117(5):1571-5.

31. Burton JH, Auble TE, Fuchs SM. Effectiveness of 50 % nitrous oxide/50% oxygen during laceration repair in children. Acad Emerg Med. 1998;5(2):112-7.

32. Lee JH, Kim K, Kim TY, Jo YH, Kim SH, Rhee JE, et al. A randomized comparison of nitrous oxide versus intravenous ketamine for laceration repair in children. Pediatr Emerg Care. 2012;28(12):1297-301.

33. Gamis AS, Knapp JF, Glenski JA. Nitrous oxide analgesia in a pediatric emergency department. Ann Emerg Med. 1989;18(2):177-81.

34. Collège des médecins du Québec. La sédation-analgésie 2015 [Available from: <http://www.cmq.org/publications-pdf/p-1-2015-04-01-fr-sedation-analgesie.pdf>.

35. Province of Ontario. Regulated Health Professions Act 1991 [Available from: <https://www.ontario.ca/laws/statute/91r18>.

36. Marinsek M, Kovacic D, Versnik D, Parasuh M, Golez S, Podbregar M. Analgesic treatment and predictors of satisfaction with analgesia in patients with acute undifferentiated abdominal pain. European journal of pain (London, England). 2007;11(7):773-8.

37. Ali S, Weingarten LE, Kircher J, et al. A survey of caregiver perspectives on children's pain management in the emergency department. CJEM. 2015;18(2):98-105.

38. Taddio A, McGrath P, Finley A. Effects of early pain experience: The human literature. Prog Pain Res Manag. 1999;13:57-74.

39. Lloyd CJ, Alredy T, Lowry JC. Intranasal midazolam as an alternative to general anaesthesia in the management of children with oral and maxillofacial trauma. Br J Oral Maxillofac Surg. 2000;38(6):593-5.

40. Lowe DA, Monuteaux MC, Ziniel S, Stack AM. Predictors of parent satisfaction in pediatric laceration repair. Acad Emerg Med. 2012;19(10):1166-72.

41. Elliott CH, Jay SM, Woody P. An observation scale for measuring children’s distress during medical procedures. J Pediatr Psychol. 1987;12(4):543–51.

42. Harman S, Zemek R, Duncan MJ, Ying Y, Petrcich W. Efficacy of pain control with topical lidocaine-epinephrine-tetracaine during laceration repair with tissue adhesive in children: a randomized controlled trial

. CMAJ. 2013;185(13):E629-34.

43. Bruns TB, Simon HK, McLario DJ, Sullivan KM, Wood RK, Anand KJ. Laceration repair using a tissue adhesive in a children's emergency department. Pediatrics. 1996;98(4):673-5.

44. Godwin SA, Burton JH, Gerardo CJ, al. e. Clinical Policy: Procedural Sedation and Analgesia in the Emergency Department. Ann Emerg Med. 2014;63(2):247-58.

45. Poonai N, Coriolano K, Klassen TP, et al., On behalf of KidsCAN PERC iPCT-SPOR (Innovative Paediatric Clinical Trials – Strategy for Patient Oriented Research) Ketodex Study Team. Adaptive randomised controlled non-inferiority multicentre trial (the Ketodex Trial) on intranasal dexmedetomidine plus ketamine for procedural sedation in children: study protocol. BMJ Open. 2020;10:e041319.

46. Yuen VM, Hui TW, Irwin MG, Yao TJ, Wong GL, MK Y. Optimal timing for the administration of intranasal dexmedetomidine for premedication in children. Anaesth. 2010;65(9):922-9.

47. Poonai N, Heath A, Ali S, Stevens H, Sabhaney V, Doyon Trottier E, et al. Optimal dose of intranasal dexmedetomidine for laceration repair in children: a phase II dose-ranging study using a Bayesian continual reassessment method. Ann Emerg Med. 2023.

48. Hartling L, Newton AS, Liang Y, et al. Music to reduce pain and distress in the pediatric emergency department: A randomized clinical trial. JAMA Pediatr. 2013;167(9):826–35.

49. Vernon DT, Schulman JL, Foley JM. Changes in children’s behavior after hospitalization. Some dimensions of response and their correlates. Am J Dis Child. 1966;111:581-93.

50. Bhatt M, Kennedy RM, Osmond MH, Krauss B, McAllister JD, Ansermino JM, et al. Consensus-based recommendations for standardizing terminology and reporting adverse events for emergency department procedural sedation and analgesia in children. Ann Emerg Med. 2009;53(4):426-35.e4.

51. Lombart B, Annequin D, Cimerman P. A simple tool to measure procedural restraint intensity in children:

validation of the PRIC (Procedural Restraint Intensity in Children) scale

. Heliyon.5:e02218.

52. Manworren RCB, Hynan LS. Clinical validation of FLACC: preverbal patient pain scale. Pediatr Nurs. 2003;29(2):140-6.

53. Smith RW, Shah V, Goldman RD, Taddio A. Caregivers' responses to pain in their children in the emergency department. Arch Pediatr Adolesc Med. 2007;161(6):578-82.

54. Zsido AN, Teleki SA, Csokasi K, Rozsa S, Bandi SA. Development of the short version of the spielberger state-trait anxiety inventory. Psychiatry Res. 2020;291:113223.

55. Harris PA, Taylor R, Thielke R, Payne J, Gonzalez N, Conde JG. Research electronic data capture (REDCap)—A metadata-driven methodology and workflow process for providing translational research informatics support. J Biomed Inform. 2009;42(2):377-81.

56. Bartley EJ, Fillingim RB. Sex differences in pain: A brief review of clinical and experimental findings. Br J Anaesth. 2013;111(1):52-8.

57. Graham BM, Weiner S, Li SH. Gender differences in avoidance and repetitive negative thinking following symptom provocation in men and women with spider phobia. Br J Clin Psychol. 2020;59(4):565-77.

58. Alabas OA, Tashani OA, Tabasam G, Johnson MI. Gender role affects experimental pain responses: A systematic review with meta‐analysis. Eur J Pain. 2012;16(9):1211-23.

59. Canada’s Drug and Technology Agency (CADTH). CADTH Methods and Guidelines: Guidelines for the Economic Evaluation of Health Technologies: Canada (4th Edition). 2017.

60. Philips Z, Bojke L, Sculpher M, Claxton K, Golder S. Good practice guidelines for decision-analytic modelling in health technology assessment. Pharmacoeconomics. 2006;24(4):355-71.

61. Petrou S. Economic evaluation in child health: Oxford University Press; 2010.

62. Baio G. Bayesian methods in health economics. Boca Raton: CRC Press; 2013.

63. Briggs A, Sculpher M, Claxton K. Decision modelling for health economic evaluation: Oxford; 2006.

64. Husereau D, Drummond M, Augustovski F, de Bekker-Grob E, Briggs AH, Carswell C, et al. Consolidated Health Economic Evaluation Reporting Standards 2022 (CHEERS 2022) statement: updated reporting guidance for health economic evaluations. Value Health. 2022;25(1):3-9.

65. Bhatt M KR, Osmond MH, Krauss B, McAllister JD, Ansermino JM, Evered LM, Roback M. Consensus Panel on Sedation Research of Pediatric Emergency Research Canada and the Pediatric Emergency Care Applied Research Network. Consensus-based recommendations for standardizing terminology and reporting adverse events for emergency department procedural sedation and analgesia in children. Ann Emerg Med. 2009;53(4):426-35.e4.

66. Barwick M. Knowledge translation planning template: The Hospital for Sick Children; 2019 [Available from: <https://www.sickkids.ca/contentassets/4ba06697e24946439d1d6187ddcb7def/79482-ktplanningtemplate.pdf>.

67. Naveen Poonai. Skeptics Guide to Emergency Medicine [Internet]; 2019. Podcast. Available from: <https://thesgem.com/2019/12/sgem278-seen-your-video-for-acute-otitis-media-discharge-instructions/>

68. Children’’s Healthcare Canada (CHC). Acute Presenting Pain Toolkit 2017 [Available from: <https://ken.childrenshealthcarecanada.ca/xwiki/bin/view/Paediatric+Pain/Acute+Presenting+Pain+Toolkit>.

69. Translating Emergency Knowledge for Kids (TREKK). 2021 [Available from: <https://trekk.ca/>.

70. Salanti G, Ades AE, Ioannidis JP. Graphical methods and numerical summaries for presenting results from multiple-treatment meta-analysis: an overview and tutorial. Journal of clinical epidemiology. 2011 Feb 1;64(2):163-71.

71. Johnson Laurel L, Bradley Susan J, Birkenfeld-Adams Andrea S, Radzins Kuksis Myra A, Maing Dianne M, Mitchell Janet N, Zucker Kenneth J. A Parent-Report Gender Identity Questionnaire for Children. 2003 Jun 2; 33(2):105-16.

72. <https://www.cihi.ca/sites/default/files/document/guidance-and-standards-for-race-based-and-indigenous-identity-data-en.pdf>

1. **Appendix**
   1. **Appendix I: Letter of Information and Consent form (as a separate document)**
   2. **Appendix I: Assent form (as a separate document)**
   3. **Appendix III: Unexpected Adverse Events and Definitions**

| **Adverse Events** | **Definitions** |
| --- | --- |
| 1. **Oxygenation** |  |
| **1.1 Oxygen Desaturation** | Oxygen desaturation AND one or more intervention(s) are performed with the intention of improving the saturation |
|  |  |
| 1. **Ventilation** |  |
| **2.1 Apnea: central** | Cessation of spontaneous respirations considered to be significant by observers and recorded as such, abnormal oxygen saturation (decrease in oxygen saturation to 90% at any point), or laryngospasm (stridor or other evidence of airway obstruction that did not improve with airway alignment maneuvers). |
| **2.2 Apnea: Obstructive** |  |
| **2.2.1 Partial Upper Airway Obstruction** | Manifested by stridor, snoring OR chest wall and suprasternal retractions AND one or more intervention(s) are performed with the intention of relieving the partial airway obstruction. |
| **2.2.2 Apnea: Complete Upper Airway Obstruction** | Ventilatory effort with NO air exchange manifested by absence of upper airway (e.g. stridor, hypoventilation, or oxygen desaturation that resolved  with repositioning of the airway) and breath sounds on auscultation *and* a loss of CO2 waveform if capnography is used AND the obstruction is relieved by one or more intervention(s) performed with the intention of relieving the complete airway obstruction. |
| **2.3** **Apnea: Laryngospasm** | Partial or complete upper airway obstruction, *with* oxygen desaturation due to involuntary and sustained closure of the vocal cords AND is NOT relieved by routine airway repositioning maneuvers, suctioning or insertion of a nasal or oral airway |
|  |  |
| 1. **Clinically Apparent Pulmonary Aspiration** | Suspicion OR confirmation of oropharyngeal or gastric contents in the trachea during the Sedation or Physiologic Recovery phase AND the appearance of respiratory signs and symptoms that were not present prior to the sedation. The new signs and symptoms must present before the end of the ED Recovery phase. The patient must develop one or more sign or symptom in any of the following three categories:   - Physical Signs: cough, crackles/rales, decreased breath sounds, tachypnea, wheezing, rhonchi OR respiratory distress - Oxygen Requirement: decrease in oxygen saturation from baseline requiring supplemental oxygen - Chest X-Ray Findings: focal infiltrate, consolidation or atelectasis |
| 1. **Retching / Vomiting** | The motor reflex response characteristic of retching with or without expulsion of gastric contents through the mouth or nose that occur during Sedation, ED Recovery or Post-Discharge phases of sedation |
| 1. **Cardiovascular Events** |  |
| **5.1 Bradycardia** | Heart rate less than 2 standards deviations below normal for age described by AHA in the PALS provider manual during the Sedation or Physiologic Recovery phase AND one or more intervention(s) are performed with the intention of improving the heart rate and cardiac output |
| **5.2 Hypotension** | Systolic blood pressure less than the 5^th^ percentile for age defined by AHA in PALS during the Sedation or Physiologic Recovery phase AND one or more one or more intervention(s) are performed with the intention of improving the blood pressure |
| 1. **Excitatory Movements** |  |
| **6.1 Myoclonus** | Involuntary, brief contraction of some muscle fibers, of a whole muscle, or of different muscles of one group, leading to movements of the corresponding body parts, usually not longer than 1/10^th^ of a second (100 milliseconds) AND interferes with the procedure, requiring a intervention or administration of medications. Hiccupping is a form of myoclonus. |
| **6.2 Muscle Rigidity** | Involuntary muscle stiffening in extension that can be associated with shaking AND interferes with the procedure, requiring an intervention or administration of medications |
| **6.3 Generalized Motor Seizure** | Temporary abnormal neural electro-physiologic phenomenon that manifests as involuntary contractions or series of contractions of the voluntary muscles. The contractions can be sustained (tonic) or repeated (tonic-clonic) |
| 1. **Response to Sedation** |  |
| **7.1 Paradoxical Response to Sedation** | Unanticipated restless or agitation in response to the administration of sedation drugs occurring during the Sedation phase AND results in the unplanned administration of reversal agents or alternative sedation drugs, a delay in the completion of the procedure or discontinuation of the procedure |
| **7.2 Unpleasant Recovery Reaction** | Abnormal patient behaviour during the ED Recovery phase that requires additional treatment ad a change or delay in patient discharge from ED. The behaviour include one or more of the following:   - Crying – inconsolable - Agitation – restless, continuous activity - Delirium – state of severe confusion, altered mental status - Hallucinations – responds to sensory (i.e. seeing, hearing or feeling) phenomena that are not physically present - Dysphoria – mood pf restlessness, depression and anxiety - Nightmares – unpleasant dreams |
| 1. **Permanent Complications** |  |
| **8.1 Permanent Neurologic Injury** | A neurologic deficit that was not present prior to sedation and does not resolve |
| **8.2 Death** | The irreversible cessation of cerebral function and spontaneous function of the respiratory and circulatory systems |
| 1. **Other** | Any effects of sedation not specifically mentioned that results in an unexpected intervention should be described and documented |
