# Supplementary material for: Anxiolysis for laceration repair in children: study protocol for an open-label multicenter adaptive trial (ALICE)
Source: PLoS One. 2025 Jun 4;20(6):e0324515. doi: 10.1371/journal.pone.0324515 (PMC12136299; doi:10.1371/journal.pone.0324515)
Supplement: S4 File — (DOCX) [file pone.0324515.s004.docx]

**Supplement 4. Informed Consent and Assent Forms**

**Informed Consent Form for Participation in a Research Study**

**Study Title**: Anxiolysis for laceration repair in children: An open-label multicenter adaptive trial (ALICE)

**Study Doctor**: Dr. Naveen Poonai, Division of Paediatric Emergency Medicine,

**Phone number**: 519-685-8500 x 58134

**Emergency contact number:** If you have developed any concerns related to your health after enrolling in the study, go directly to the emergency department or call 911. For questions that are not urgent, please contact the research team at (519) 685-8500 x 56174 or email: vinolia.arthurhayward@lhsc.on.ca

**Sponsor/Funder(s):** Dr. Naveen Poonai, Canadian Institutes of Health Research, Academic Medical Association of Southwestern Ontario, Western Strategic Support for CIHR Success and London Health Sciences Center.

Contact numbers and information are noted at the end of this document under the section heading “Contacts”.

**INTRODUCTION**

*As a Substitute Decision Maker, you are being asked to provide informed consent on behalf of a person who is unable to provide consent for him/herself. If the participant gains the capacity to consent for him/herself, your consent for them will end. Throughout this form, “you” means the person you are representing.*

You are being invited to participate in a clinical trial that is looking for the best way to make repair of a cut in the skin (laceration) less distressing. You are invited to participate in this trial because you require repair of your laceration and are between 2 and 12 years old. Your caregiver will also be invited to rate their anxiety during your visit. Although we will use an anesthetic applied to the surface of your cut, many children still experience anxiety and fear during laceration repair. Your caregiver, nurse, and doctor believe that you may experience anxiety and fear during your laceration and providing you with an anxiolytic (a medication to help you feel calm) will help reduce your distress during this procedure. To do this, we will use one of the three medications: dexmedetomidine or midazolam given using a nasal spray (intranasal) or nitrous oxide given using a face mask. These drugs have all been used to sedate children and have an excellent safety record. We just don’t know what the best medication is. This is important because many children undergo laceration repair and experience distress. Our results may be used to help these children have a better healthcare experience. This consent form provides you with what you need to make an informed decision to participate. Please read this document carefully and feel free to ask any questions. All your questions should be answered to your satisfaction before you decide whether to participate in this research study.

**IS THERE A CONFLICT OF INTEREST?**

There are no conflicts of interest to declare related to this study. If you would like additional information about the funding for this study, or about the role of the doctor in charge of this study, please speak to the study staff or the **Patient Relations Office at LHSC at (519) 685-8500 ext. 52036**

**WHAT MEDICATIONS WILL BE USED IN THIS STUDY?**

We will use one of three anxiolytics to repair your laceration: intranasal dexmedetomidine, intranasal midazolam, or inhaled nitrous oxide. Intranasal dexmedetomidine and midazolam have been used for many procedures in children such as dental work, MRIs, lumbar punctures, laceration repair, and for relaxing patients before surgery. You will require up to two pairs of nasal sprays, depending on your weight. Each spray contains no more than 0.5 mL of fluid. We use a small amount to minimize discomfort and maximize the ability of the medication to provide anxiolysis. Each pair of sprays will be separated by at least a minute. This method of giving medication using nasal sprays has been used before and is generally well tolerated in children. Nitrous oxide is commonly used for dental work and is a tasteless, odorless gas given using a face mask. However, we still don’t know which drug is the best for providing anxiolysis to decrease distress during laceration repair in children. The study medications are regulated by Health Canada who has approved their use for this trial.

In keeping with routine care, you will receive an anesthetic called lidocaine-epinephrine-tetracaine (LET) on the surface of your laceration. This anesthetic takes 30 minutes to work. While you are waiting, the study medication will be given. Afterwards, your doctor will repair your laceration. It is important to know that you may not be fully asleep while your laceration is being repaired because we are using an anxiolytic rather than a general anesthetic.

**WHY IS THIS STUDY BEING DONE?**

The distress caused by laceration repair can negatively affect the length of time it takes to perform the procedure, your reaction to future medical procedures, the cosmetic results, and your satisfaction with your healthcare experience. The purpose of this study is to find out which anxiolytic is to best to reduce distress during laceration repair. We anticipate that our findings will result in an emergency department experience that is easier, faster, less invasive, less painful, and significantly less distressing for you and your caregiver.

**WHAT HAPPENS IF YOU CHOOSE NOT TO PARTICIPATE?**

Participating in this study is optional. If you choose not to participate, it will not affect your care in the emergency department or your relationship with the healthcare staff. You will still receive LET as an anesthetic but will not typically receive any anxiolytic.

**HOW MANY CHILDREN WILL TAKE PART IN THIS STUDY?**

This study will take place across four paediatric emergency departments in Canada (Children’s Hospital, London Health Sciences Centre, London; Stollery Children’s Hospital, Edmonton; Ste Justine, Montreal; BC Children’s, Vancouver). The study will recruit a total of 300 children, and we estimate it will take two years to complete. The results should be known within three years. At our site, Children’s Hospital, London Health Sciences Centre, London we expect to include at least 60 participants.

**WHAT WILL HAPPEN DURING THIS STUDY?**

If your laceration requires repair using stitches based on the opinion of your doctor, LET will be placed on your wound. A research assistant will explain the study procedures in detail, answer any questions or concerns you may have, and seek informed consent (and assent when appropriate).

If you agree to participate, you will be assigned at random (like the flip of a coin) to one of three groups: intranasal dexmedetomidine, intranasal midazolam, or inhaled nitrous oxide. Regardless of what group you are assigned, everyone will receive an anxiolytic. The intranasal medications will be given shortly after the anesthetic LET is placed and the nitrous oxide will be given -three minutes before your procedure. Everyone on the healthcare team will know what medication you are receiving but will not be able to choose to which group you will be assigned.

We are mainly interested in knowing how much distress (if any) you experience when receiving one of the three interventions. You will be asked to score your experience after receiving the drugs and some medical information will be obtained directly from you or from your medical chart. We are also interested in knowing how long you are asleep (if at all), length of stay in the emergency department, side effects, and your satisfaction with your laceration repair experience.

Another research assistant who is not in the emergency department (outcome assessor) will score your level of distress during laceration repair. This research personnel is from our local team (in London – LHSC) and will be selected and hired to do this task. To determine this without introducing bias, we will record a video segment of your face and body after you receive your medication. We cannot say with certainty for how long the research nurse will be recording the video but it will begin just prior to laceration repair and will stop immediately after the repair is completed. We estimate that the duration of the recording will be between 5 and 10 minutes. Your caregiver will be invited to stay right by your side for the entire time. For security purposes, the video will be recorded using a Canon VIXIA HF R700 camcorder (not equipped with WiFi or Bluetooth) and stored on an SD card. Once the video has been recorded, the research assistant will upload the video onto an online platform called Sync^TM^, a Canadian cloud-based service that is compliant with all federal and provincial data privacy guidelines. Sync’s zero-knowledge storage platform guarantees your privacy by providing end-to-end encryption and provides access only to a small group of individuals on the research team. All videos recorded and uploaded onto Sync^TM^ will be deleted when the study is complete.

All study data obtained from you apart from the video will be uploaded and stored on a secure electronic database system known as Research Electronic Data Capture (REDCap). This will allow accurate recording of study data but more importantly, ensure that your data is not accessible to anyone outside the study group.

**WHAT DATA WILL BE COLLECTED FROM YOU?**

At enrollment, we will collect some personal information from you (name, telephone number, email address, age, sex, gender, and hospital number). This information is needed to track side effects, describe the study sample, and allow us to communicate with you or your caregiver for follow-up or in case you agree to participate in future studies related to dexmedetomidine, midazolam, or nitrous oxide.

Gender Expression

We will collect from your caregiver, the Gender Identify Questionnaire for Children. This questionnaire will ask about how you present yourself as a boy or girl. You and your caregiver may choose not to answer these questions.

Nasal Irritation

The research nurse will collect information to rate pain with the nasal spray. The *Faces, Legs, Arms, Cry, Consolability* (FLACC) scale will be used to rate how much it hurts.

When you are ready for discharge, we will collect information on the onset and duration of sedation, length of time you spent in the emergency department, side effects, and satisfaction from you, your caregiver, and your healthcare staff.

Approximately 72-96 hours after discharge, the research assistant will be contacting you or your caregiver via telephone or email to see if you had any late side effects using a short survey called the Post Hospital Behavior Questionnaire. Although late side effects are uncommon, a follow-up survey will help us identify any abnormal behaviours that may be related to the anxiolytic you received such as disturbances in eating, sleeping, restlessness, or anxiety.

**CAN YOU WITHDRAW FROM PARTICIPATING IN THE STUDY ONCE YOU ARE ENROLLED?**

You can choose to end your participation in this research study at any time without having to provide a reason and without affecting the quality or timeliness of your care. However, data obtained up to time you withdraw will be retained by the study team for analysis. It is important that we collect information on participants who withdraw from the study to detect any side effects. A copy of the Letters of Information, Consent, and Assent (if applicable) will be given to you for your records. The informed consent process will be conducted and documented on REDCap (including the date) before you undergo any study-related procedure or data collection.

If you decided to withdraw while you are still in the ED, please let your research assistant or anyone in the health care team know. If you decide to withdraw after you discharge, please contact **Dr. Naveen Poonai at (519) 685-8500 x 58134 / naveen.poonai@lhsc.on.ca OR Vinolia Arthur-Hayward at (519) 685-8500 x 56174 / vinolia.arthurhayward@lhsc.on.ca.**

**CAN YOUR DOCTOR WITHDRAW YOU FROM THE STUDY ONCE YOU ARE ENROLLED?**

Your doctor or a member of the research team may stop your participation in the study prematurely, and without your consent, for the following reasons:

- The occurrence of a side effect, laboratory abnormality, or other medical condition in which continued participation in the study would be detrimental to your health
- If you are found to meet a study exclusion criterion (either newly developed or not previously recognized)

If you are removed from this study, the study doctor or member of the research team will discuss the reasons with you and plans will be made for your continued care outside of the study.

**WHAT ARE THE RISKS OR HARMS OF PARTICIPATING IN THIS STUDY?**

The medications in this study may have short-lived side effects. Intranasal dexmedetomidine may cause nausea, vomiting, and lower heart rate but this is uncommon with the intranasal route. Serious side effects such as airway obstruction are possible but rare in the doses and age range involved in this study. Intranasal midazolam may cause some temporary discomfort in the nose, drowsiness, or dizziness. Rarely, a child may become hyperactive or irritable. Nitrous oxide may cause nausea, dizziness, or vomiting. Headache and fatigue are rare but possible. Your doctor and nurse will watch you closely to see if you have any side effects and will urgently treat them if needed.

If your doctor believes that the medication is not providing sufficient anxiolysis and wishes to give you a medication other than the ones we are studying, they may do so and the choice of the medication is up to them, based on their experience and comfort level.

If you experience side effects that you believe require a hospital visit, it is important that you make every effort to return to the hospital closest to you. If you need immediate treatment and are unable to return to the hospital, you should call an ambulance and the study doctor should be contacted as soon as possible.

While there is a risk of breach of privacy and confidentiality because we are obtaining videos including facial features, we can assure you that we do our best to minimize this risk by maintaining all electronic files encrypted as soon as it is obtained.

**WHAT ARE THE REPRODUCTIVE RISKS?**

Since the safe use in pregnancy, including obstetrics (either vaginal or abdominal delivery), has not been established, patients who report that they may be pregnant will be excluded.

**WHAT ARE THE BENEFITS OF PARTICIPATING IN THIS STUDY?**

As a participant in this study, you may experience a calmer or less anxiety-provoking experience during your laceration repair. Also, it may help to improve the care of children in the future who require an anxiolytic during laceration repair.

**HOW WILL PARTICIPANT INFORMATION BE KEPT CONFIDENTIAL?**

If you decide to participate in this study, the research team will only collect the information they need for this study. All information collected will be kept confidential and will not be part of your medical record. You will be assigned a unique identification number that contains no personal identifiers. This number will appear on all the information we collect from the study. Research data will be stored electronically in REDCap at the Women and Children’s Health Research Institute (WCHRI) at the University of Alberta. The personnel at this center have the expertise to maintain quality standards to ensure your privacy. Paper forms (Letters of Information, Consent, and Assent if applicable) will be stored for 15 years according to Health Canada's policies in a locked and secure research cupboard that is accessible only to the research team. Video segments will only be accessible to a limited number of people on the research team and will be deleted at the end of the study.

The REDCap is a secure web application for building and managing online surveys and databases. This will allow accurate recording of study data but more importantly, ensure that data is not accessible to anyone outside the research team. Also, the REDCap data is not shared between participating sites. Only the main site will be able to access information from other sites and all information entered REDCap is de-identified. To have access to REDCap, each site’s research team must be authorized by the principal investigator or designated to have access and have a username and login created. Each username and login is created by an individual and not shared with a team or groups.

**WHO WILL CONDUCT MONITORING OF THE STUDY?**

Study monitoring is the action of supervising the progress of a clinical trial to ensure that the study is being conducted, recorded, and reported in accordance with the protocol and Health Canada regulations. The Maternal Infant Child and Youth Research Network (MICYRN) will be providing study monitoring on behalf of the sponsor. The Lawson Quality Assurance will be providing onsite monitoring of the study locally.

**WILL THE STUDY DATA BE ENTERED INTO A DATABASE FOR FUTURE USE?**

Following your participation, your identity will not be revealed. Any report published as a result of this study will not identify you by name. By signing the consent form, you give permission to the research team to access only personal health information that we deem necessary to conduct the research study. You are also giving permission to the following organizations to have access to study related information (including personal health information) to ensure that the study is following the proper laws and regulations.

- Dr. Naveen Poonai
- Representatives of Western University's Health Sciences Research Ethics Board,
- Health Canada (because Health Canada oversee the use of natural health products/drugs/devices in Canada) to have access to study related information (including personal health information) to ensure that the study is following the proper laws and regulations.
- Representatives of Lawson Quality Assurance (LQA) Education Program may look at study data for Quality Assurance purposes.

Information that is collected about you for the study (called study data) may also be sent to the organizations listed above. Your name, address, or other information that may directly identify you will not be used. The records received by these organizations may contain your participant code, initials, sex, date of birth, and type of intervention given during your visit to the emergency department.

A copy of the consent form that you sign to enter the study may be included in your health record/hospital chart.

**WHAT ARE THE RESPONSIBILITIES OF STUDY PARTICIPANTS?**

- Tell the research assistant about your current medical conditions, including all prescription and non-prescription medications and supplements. This is for your safety as these may interact with the study drugs.
- Tell the study doctor if you are thinking about participating in another research study.
- Tell the study doctor if you become pregnant while participating in this study.

**HOW LONG WILL YOU BE IN THIS STUDY?**

Participation in the study while you are in the emergency department will last for about one to two hours from the time you sign the Letters of Information, Consent, and Assent (if applicable) to the time you wake up from up from the anxiolytic. You will be discharged home as soon as your physician and nurse believes that you are safe to be sent home. The research assistant will contact you either by phone or email 72-96 hours after discharge to complete the follow-up survey. The survey should take approximately 10-15 minutes to complete and can be done over the phone or online.

**WILL FAMILY DOCTORS/HEALTH CARE PROVIDERS KNOW WHO IS PARTICIPATING IN THIS STUDY?**

Your family doctor or pediatrician will receive a study letter informing them that you are participating in the study. Please, provide your family physician or pediatrician information so the research team may send a letter indicating that you agreed to participate in this study.

**WILL information about this study BE available online?**

The trial’s objectives, protocol, and potential implications will be featured on the study’s website <https://alicepedsed.wordpress.com> with navigation tabs for specific stakeholders: investigators, caregivers, children, and clinicians. A detailed description of the study’s protocol will be listed on [*www.clinicaltrials.gov*](http://www.clinicaltrials.gov), an international clinical trial registry. The study’s protocol and findings will be submitted for publication in a medical journal and may be presented at international conferences and local research meetings. However, any information that can identify you such as name, address, date of birth, etc. will not appear on any website, publication, or presentation. For the reasons of transparency and education, it is strongly encouraged by many medical journals and other authorities to publish anonymized data from clinical studies for public use. Examples of anonymized data from this study include the most common location of laceration, level of pain characterized by a number, level of satisfaction with the sedation also characterized by a number, etc. While anonymized data may be retained indefinitely, as per institutional policies, only identifiable information must be deleted after the data retention period (15 years for Health Canada studies). The anonymized data is visible to researchers or the general public after the study is over. Future researchers may use anonymized data to improve knowledge about procedural sedation in children (1– 12 years) presenting to paediatric emergency departments with a laceration.

**WHAT IS THE COST TO PARTICIPANTS?**

There are no costs to participation in this study.

**ARE STUDY PARTICIPANTS PAID TO BE IN THIS STUDY?**

If you decide to participate in this study, you will be offered a token of appreciation in the amount of $50 that will be mailed to your home after discharge from the emergency department.

**WHAT ARE THE RIGHTS OF PARTICIPANTS IN A RESEARCH STUDY?**

You have the right to be informed of the results of this study once the entire study is complete. A summary of the results will be publicly available at [*http://www.clinicaltrials.gov*](http://www.clinicaltrials.gov)*.*

Your rights to privacy are legally protected by Canadian federal and provincial laws that require safeguards to ensure that your privacy is respected.

The rights and welfare of your participation will be protected by emphasizing to you that the quality of your medical care will not be adversely affected if you decline to participate in this study. Furthermore, by signing this form you do not give up any of your legal rights against the study doctor, sponsor or involved institutions for compensation, nor does this form relieve the study doctor, sponsor or their agents of their legal and professional responsibilities.

You will be given a copy of this signed and dated consent form prior to participating in this study.

**WHOM DO PARTICIPANTS CONTACT FOR QUESTIONS?**

If you have any questions or concerns, feel free to contact at any time:

- Dr. Naveen Poonai at London Health Sciences Centre at (519) 685-8500 x 58134 - Naveen.Poonai@lhsc.on.ca
- Vinolia Arthur-Hayward Children’s Hospital, London Health Sciences Centre at (519) 685-8500 x 56174 - vinolia.arthurhayward@lhsc.on.ca
- The Office of Human Research Ethics (519) 661-3036, 1-844-720-9816, email:[ethics@uwo.ca](mailto:ethics@uwo.ca).

If you have any questions about your rights as a research participant or the conduct of this study, you may contact the Patient Relations Office at LHSC at (519) 685-8500 ext. 52036

You will receive a copy of this information form after enrolling in the study.

**CONSENT FORM**

Anxiolysis for laceration repair in children: a multicenter adaptive randomized trial (ALICE)

**SIGNATURES**

- All of my questions have been answered.
- I understand the information within this informed consent form.
- I allow access to my medical records as explained in this consent form.
- I do not give up any of my legal rights by signing this consent form.
- I agree or agree to allow the person I am responsible for, to take part in this study.
- I understand that if I do not agree, I do not need to participate.

**I have read the Letter of Information, have had the nature of the study explained to me and I agree to participate. All questions have been answered to my satisfaction.**

**May we contact you for future studies related to laceration repair and to this study?**

Please indicate by checking the Yes **OR** NO boxes:

🞎 Yes, I agree to be contacted for future research studies_______ (ADD INITIALS)

🞎 No, I do NOT agree to be contacted for future research studies_________ (ADD INITIALS)

___________________________ _______________________ _________

Substitute Decision Maker PRINTED NAME Date

**___________________________**  **_______________________** **_________**

Participant Signature PRINTED NAME Date

*“My signature means that I have explained the study to the participant named above. I have answered all questions.”*

____________________________ ______________________ _________________

Signature of Person Conducting PRINTED NAME & ROLE Date the Consent Discussion

Complete the following section only if the participant is unable to read or requires an oral translation:

If the participant is assisted during the consent process, please check the relevant box and complete the signature space below:

The person signing below acted as an interpreter, and attests that the study as set out in the consent form was accurately sight translated and/or interpreted, and that interpretation was provided on questions, responses and additional discussion arising from this process.

Note: Family members and healthcare workers are not allowed to act as a partial witness

____________________________ ______________________ _________________

Signature of Impartial PRINTED NAME Date

Translator

*(If participant was unable to read/required an oral translation)*

Language: *________________________________*

The consent form was read to the participant. The person signing below attests that the study as set out in this form was accurately explained to the participant, and any questions have been answered.

____________________________ _______________________ _________________

PRINT NAME Signature Date

of witness

____________________________

Relationship to Participant

Please note: More information regarding the assistance provided during the consent process should be noted in the medical record for the participant if applicable, noting the role or relationship of the impartial witness.

**Assent Letter to Participate**

**We understand that a child providing assent will depend on the capacity of each individual (child) to understand what is explained and the nature of the study itself. Considering these two relevant points, we expect to obtain assent from children between 7 to 12 years of age. However, we will take into account that a younger child (5 to 6 years of age) could be mature enough to comprehend and/or read the assent form. These children will be encouraged to sign the assent form as well.**

Study Title: Anxiolysis for laceration repair in children: An open-label multicenter adaptive trial (ALICE)

**Principal Investigator:** Dr. Naveen Poonai, Paediatric Emergency Physician, Children’s Hospital, London Health Sciences Centre (LHSC)

**Protocol number: REB#120985**

**Sponsor:** Dr. Naveen Poonai

**Why are we doing this study?**

You have a cut that needs to be fixed. Usually, we give you a numbing medicine that is placed over your cut using a cotton ball to reduce some of the pain. However, many kids still feel upset or scared when doctors fix their cuts, even though they don’t feel much pain, and need some help to feel calm. Your caregiver, nurse, and doctor believe that you may need some help to feel calm while we fix your cut. This is a science study, and the doctors want to find the safest and best medicine to help you feel calm. We are studying three different medicines to help you feel calm. Two of the medicines are sniffed in using sprays in the nose and one is breathed using either a facemask that fits gently over your mouth and nose that you control. All of the medicines are very safe in kids with cuts just like yours and may make you sleep or feel like you are falling sleep. We would like to see if you would be interested in joining our science study to help us find the best medicine.

**What will happen during this study?**

1. You will receive a numbing medicine to help with the pain of your cut.

2. You will receive a medicine that is either sniffed in using a spray in your nose or breathed in using a facemask. We do not plan to use any needles to help you feel calm.

3. We will be making a video of you while your cut is being fixed so that we can find out how calm the medicine has made you. We will not share the video with anyone else but the science team. This may help us to learn a better way to help other kids with a cut like yours. The video will be stored on a database called Sync.com and then moved to a computer protected with a secret password and only available to the research team. This computer will be kept in the office of the doctor responsible for this science study for 15 years. After that time, all information about this science study will be deleted from the computer’s memory, including the videos and other information.

4. Your caregiver will be asked to stay right beside you the entire time. After your cut is fixed, your caregiver will help you answer a few questions about how you felt.

**Will it hurt?**

We will give you a pain medicine using a cotton ball over your cut to reduce pain while your cut is being fixed. The medicine sprayed in your nose will not hurt but may feel a little funny. The medicine you breathe in will not hurt.

**Do you have to be in this study?**

You do not have to be in this study if you don’t want to. You will still be cared for in the best possible way.

**Who you should contact if you have questions?**

You can ask questions at any time now or later. If you have any questions, please talk to anyone on the research team, your caregiver or the doctor or nurse who is taking care of you.

You can also contact:

- **Dr. Naveen Poonai** at the Children’s Hospital, London Health Sciences Centre at **(519) 685-8500 x 58134 - Naveen.Poonai@lhsc.on.ca**

OR

- **Vinolia Arthur Hayward** Children’s Hospital, London Health Sciences Centre at **(519) 685-8500 x 56174** **-** [vinolia.arthurhayward@lhsc.on.ca](mailto:vinolia.arthurhayward@lhsc.on.ca)

If you have any questions about your rights as a research participant or the conduct of this study, you may contact the Patient Relations Office at LHSC at (519) 685-8500 ext. 52036

**Assent Form**

Title: Anxiolysis for laceration repair in children: A multicenter adaptive randomized trial (ALICE)

Assent:

I have read this letter and I want to participate in this study.

___________________ ________________________ ____________

Child Name (Printed) Child Signature Date

Due to injury or any other temporary limitation, the child was not able to provide written assent, but verbal assent was obtained

___________________ ________________________ ____________

Person Obtaining Signature of Person Obtaining Date

Assent (Printed) Assent
