# Supplementary material for: Anxiolysis for laceration repair in children: study protocol for an open-label multicenter adaptive trial (ALICE)
Source: PLoS One. 2025 Jun 4;20(6):e0324515. doi: 10.1371/journal.pone.0324515 (PMC12136299; doi:10.1371/journal.pone.0324515)
Supplement: S5 File — (DOCX) [file pone.0324515.s005.docx]

**Supplement 5. Unexpected Adverse Events and Definitions**

| **Adverse Events** | **Definitions** |
| --- | --- |
| 1. **Oxygenation** |  |
| **1.1 Oxygen Desaturation** | Oxygen desaturation AND one or more intervention(s) are performed with the intention of improving the saturation |
|  |  |
| 1. **Ventilation** |  |
| **2.1 Apnea: central** | Cessation of spontaneous respirations considered to be significant by observers and recorded as such, abnormal oxygen saturation (decrease in oxygen saturation to 90% at any point), or laryngospasm (stridor or other evidence of airway obstruction that did not improve with airway alignment maneuvers). |
| **2.2 Apnea: Obstructive** |  |
| **2.2.1 Partial Upper Airway Obstruction** | Manifested by stridor, snoring OR chest wall and suprasternal retractions AND one or more intervention(s) are performed with the intention of relieving the partial airway obstruction. |
| **2.2.2 Apnea: Complete Upper Airway Obstruction** | Ventilatory effort with NO air exchange manifested by absence of upper airway (e.g. stridor, hypoventilation, or oxygen desaturation that resolved  with repositioning of the airway) and breath sounds on auscultation *and* a loss of CO2 waveform if capnography is used AND the obstruction is relieved by one or more intervention(s) performed with the intention of relieving the complete airway obstruction. |
| **2.3** **Apnea: Laryngospasm** | Partial or complete upper airway obstruction, *with* oxygen desaturation due to involuntary and sustained closure of the vocal cords AND is NOT relieved by routine airway repositioning maneuvers, suctioning or insertion of a nasal or oral airway |
|  |  |
| 1. **Clinically Apparent Pulmonary Aspiration** | Suspicion OR confirmation of oropharyngeal or gastric contents in the trachea during the Sedation or Physiologic Recovery phase AND the appearance of respiratory signs and symptoms that were not present prior to the sedation. The new signs and symptoms must present before the end of the ED Recovery phase. The patient must develop one or more sign or symptom in any of the following three categories:   - Physical Signs: cough, crackles/rales, decreased breath sounds, tachypnea, wheezing, rhonchi OR respiratory distress - Oxygen Requirement: decrease in oxygen saturation from baseline requiring supplemental oxygen - Chest X-Ray Findings: focal infiltrate, consolidation or atelectasis |
| 1. **Retching / Vomiting** | The motor reflex response characteristic of retching with or without expulsion of gastric contents through the mouth or nose that occur during Sedation, ED Recovery or Post-Discharge phases of sedation |
| 1. **Cardiovascular Events** |  |
| **5.1 Bradycardia** | Heart rate less than 2 standards deviations below normal for age described by AHA in the PALS provider manual during the Sedation or Physiologic Recovery phase AND one or more intervention(s) are performed with the intention of improving the heart rate and cardiac output |
| **5.2 Hypotension** | Systolic blood pressure less than the 5^th^ percentile for age defined by AHA in PALS during the Sedation or Physiologic Recovery phase AND one or more one or more intervention(s) are performed with the intention of improving the blood pressure |
| 1. **Excitatory Movements** |  |
| **6.1 Myoclonus** | Involuntary, brief contraction of some muscle fibers, of a whole muscle, or of different muscles of one group, leading to movements of the corresponding body parts, usually not longer than 1/10^th^ of a second (100 milliseconds) AND interferes with the procedure, requiring a intervention or administration of medications. Hiccupping is a form of myoclonus. |
| **6.2 Muscle Rigidity** | Involuntary muscle stiffening in extension that can be associated with shaking AND interferes with the procedure, requiring an intervention or administration of medications |
| **6.3 Generalized Motor Seizure** | Temporary abnormal neural electro-physiologic phenomenon that manifests as involuntary contractions or series of contractions of the voluntary muscles. The contractions can be sustained (tonic) or repeated (tonic-clonic) |
| 1. **Response to Sedation** |  |
| **7.1 Paradoxical Response to Sedation** | Unanticipated restless or agitation in response to the administration of sedation drugs occurring during the Sedation phase AND results in the unplanned administration of reversal agents or alternative sedation drugs, a delay in the completion of the procedure or discontinuation of the procedure |
| **7.2 Unpleasant Recovery Reaction** | Abnormal patient behaviour during the ED Recovery phase that requires additional treatment ad a change or delay in patient discharge from ED. The behaviour include one or more of the following:   - Crying – inconsolable - Agitation – restless, continuous activity - Delirium – state of severe confusion, altered mental status - Hallucinations – responds to sensory (i.e. seeing, hearing or feeling) phenomena that are not physically present - Dysphoria – mood pf restlessness, depression and anxiety - Nightmares – unpleasant dreams |
| 1. **Permanent Complications** |  |
| **8.1 Permanent Neurologic Injury** | A neurologic deficit that was not present prior to sedation and does not resolve |
| **8.2 Death** | The irreversible cessation of cerebral function and spontaneous function of the respiratory and circulatory systems |
| 1. **Other** | Any effects of sedation not specifically mentioned that results in an unexpected intervention should be described and documented |
