# Supplementary material for: Anxiolysis for laceration repair in children: study protocol for an open-label multicenter adaptive trial (ALICE)
Source: PLoS One. 2025 Jun 4;20(6):e0324515. doi: 10.1371/journal.pone.0324515 (PMC12136299; doi:10.1371/journal.pone.0324515)
Supplement: S6 File — (PDF) [file pone.0324515.s006.pdf]

Approval Form

Date:	Tuesday, March 11, 2025
Principal Investigator:	Samina Ali
Study ID:	Pro00145495
Study Title:	Anxiolysis for laceration repair in children: An open-label multicenter adaptive trial (ALICE)
Protocol Number:	ALICE2022
Approval Expiry Date:	Tuesday, March 10, 2026
Funding/Sponsor:	Accelerating Clinical Trials (ACT)<br>Academic Medical Organization of South Western Ontario (AMOSO)<br>Western Strategic Support for Success (WSSS)<br>New Frontiers in Research Fund (NFRF)

Thank you for submitting the above study to the Health Research Ethics Board - Biomedical Panel, which was reviewed by the Full Board at the January 8, 2025 meeting. All issues arising from the meeting have been addressed. The study is now approved. The following documentation forms part of this approval:

Approved Documents:

Consent Forms

ALICE_Consent Form_11Mar2025_clean.docx

ALICE_Health care provider LOI_13Feb2025.docx

Assent Forms

ALICE_Assent Form_OLDER CHILD_17Jan2025_clean.doc

ALICE_Assent Form_YOUNGER CHILD_17Jan2025_clean.doc

Questionnaires, Cover Letters, Surveys, Tests, Interview Scripts, etc.

CRF ALICE_19Jul2024.docx

Protocol/Research Proposal

ALICE Protocol_v2.9_12Sep2024.docx

Investigator Brochures/Product Monographs

Nitrous Oxide Compressed_Air Liquide.pdf

Dexmedetomidine PM-Auro.pdf

Midazolam PM-Pfizer.pdf

Health Canada No Objection Letter

20Mar2024_NOL284984.pdf

Other Documents

ALICE_email script_14Jan2024.docx

Schedule B_ALICE STUDY Budget Template-Lead site12Aug2024.xlsx

The Health Research Ethics Board assessed all matters required by section 50(1)(a) of the Health Information Act. Subject consent for access to identifiable health information is required for the research described in the ethics application, and appropriate procedures for such consent have been approved by the HREB - Biomedical Panel. In order to comply with the Health Information Act, a copy of the approval form is being sent to the Office of the Information and Privacy Commissioner.

Any proposed changes to the study must be submitted to the REB for approval prior to implementation. A renewal report must be submitted next year prior to the expiry of this approval if your study still requires ethics approval. If you do not

renew on or before the renewal expiry date (Tuesday, March 10, 2026), you will have to re-submit an ethics application.

The membership of the Health Research Ethics Board - Biomedical Panel complies with the membership requirements for research ethics boards as defined in Division 5 of the Food and Drug Regulations and the Tri Council Policy Statement. The HREB - Biomedical Panel carries out its functions in a manner consistent with Good Clinical Practices.

Approval by the REB does not constitute authorization to initiate the conduct of this research. The Principal Investigator is responsible for ensuring required approvals from other involved organizations (e.g., Alberta Health Services, Covenant Health, community organizations, school boards) are obtained, before the research begins.

Sincerely,

Dr. Glen J. Pearson, BSc, BScPhm, PharmD, FCSHP, FCCS Chair, Health Research Ethics Board – Biomedical Panel

Note: This correspondence includes an electronic signature (validation and approval via an online system).
