# Supplementary material for: Anxiolysis for laceration repair in children: study protocol for an open-label multicenter adaptive trial (ALICE)
Source: PLoS One. 2025 Jun 4;20(6):e0324515. doi: 10.1371/journal.pone.0324515 (PMC12136299; doi:10.1371/journal.pone.0324515)
Supplement: S7 File — (PDF) [file pone.0324515.s007.pdf]

CHU Sainte-Justine
Le centre hospitalier universitaire mère-enfant

Université de Montréal

Le 5 mars 2025

Docteure Evelyne Doyon-Trottier
CHU Sainte-Justine

Objet	Approbation éthique initiale - CÉR
	2025-7927 Anxiolytique pour la réparation des lacérations chez les enfants : un essai adaptatif multicentrique ouvert (ALICE)<br><br>Co-chercheur : Jocelyn Gravel

Bonjour,

Votre projet pourra commencer dans nos murs uniquement lorsque la personne mandatée au CHU Sainte-Justine aura émis et déposé son autorisation pour la réalisation du projet de recherche au CHU Sainte-Justine.

Le Comité d'éthique de la recherche du CHU Sainte-Justine, à sa réunion plénière tenue le 12 décembre 2024, a évalué le projet mentionné en rubrique. Suite à vos réponses satisfaisantes, le Comité accorde son approbation éthique en date du 5 mars 2025.

L'examen scientifique a été réalisé par AMOSO et AEC (Accélérer les Essais Cliniques - Consortium canadien).

Les documents suivants ont été approuvés :

- Protocole de recherche, version 2.8, daté du 19 juillet 2024
- Formulaire d'information et de consentement daté du 20 janvier 2025 (version française)
- Formulaire d'information et de consentement daté du 17 février 2025 (version anglaise)
- CRF et questionnaires, version 2.7, daté du 4 avril 2024

Les formulaires d'information et de consentement estampillés ont été déposés dans le dossier du projet. Nous vous prions de vous servir de ces versions estampillées.

Nous avons également pris connaissance des documents ci-dessous :

- Safety Data Sheet Nitrous Oxide (Compressed), version 1.0, daté du 15 mai 2017
- Product monograph including patient medication information Midazolam Injection USP daté du 3 janvier 2023
- Product monograph including patient medication information PrDexmedetomidine Hydrochloride for Injection daté du 27 novembre 2023
- Pharmacy manual, version 1.1, daté du 14 février 2024

Tous les projets de recherche impliquant des sujets humains doivent être réévalués annuellement. La durée de votre approbation sera effective jusqu'au **5 mars 2026**. Il est de votre responsabilité de soumettre une demande au comité pour que l'approbation éthique soit renouvelée avant la date d'expiration. Il est également de votre responsabilité d'aviser le comité dans les plus brefs délais de toute modification au projet et/ou de tout événement grave et inattendu susceptible d'augmenter le niveau de risque ou d'influer sur le bien-être du participant.

Considérez que pour une collaboration avec un tiers impliquant des transferts de fonds ou de données/matériel biologique, une entente (contrat) est nécessaire. Celle-ci doit être gérée par le Bureau des ententes de recherche.

À noter que :

- Le Comité d'éthique de la recherche du CHU Sainte-Justine (numéro FWA00021692) est désigné par le gouvernement du Québec (MSSS).
- La composition de ce comité d'éthique pour la recherche satisfait aux exigences pertinentes prévues dans le titre 5 de la partie C du Règlement sur les aliments et drogues.
- Le comité d'éthique de la recherche exerce ses activités d'une manière conforme aux Bonnes pratiques cliniques, à l'Énoncé de politique des trois conseils : Éthique de la recherche avec des êtres humains, au Plan d'action ministériel en éthique de la recherche et en intégrité scientifique, aux lois et règlements applicables au Québec et au Canada, ainsi qu'aux standards américains énoncés par le Code of Federal Regulations.

En vous souhaitant du succès dans la réalisation de votre projet,

Marie-Hélène La France
Responsable administrative des nouveaux projets de recherche
Bureau de l'éthique de la recherche
pour Patrick Gogognon, conseiller en éthique et vice-président du Comité d'éthique de la recherche

Signé le 2025-03-05 à 15:11
