# Supplementary material for: Anxiolysis for laceration repair in children: study protocol for an open-label multicenter adaptive trial (ALICE)
Source: PLoS One. 2025 Jun 4;20(6):e0324515. doi: 10.1371/journal.pone.0324515 (PMC12136299; doi:10.1371/journal.pone.0324515)
Supplement: S8 File — (PDF) [file pone.0324515.s008.pdf]

UBC C&W Research Ethics Board
A2-141A, 950 West 28th Avenue
Vancouver, BC V5Z 4H4
Tel: (604) 875-3103 Fax: (604) 875-2496
Email: cwreb@bcchr.ubc.ca
Website:
http://www.phsa.ca/researcher/ethics-approvals/research-ethics-approval

ETHICS CERTIFICATE OF FULL BOARD APPROVAL

PRINCIPAL INVESTIGATOR:	INSTITUTION / DEPARTMENT:	UBC C&W NUMBER:
Vikram J. Sabhaney	UBC/Medicine, Faculty of/Paediatrics	H24-02187

INSTITUTION(S) WHERE RESEARCH WILL BE CARRIED OUT:

Institution	Site
BC Children's Hospital (includes Research Institute)	BC Children's Hospital (includes Research Institute)

Other locations where the research will be conducted:
N/A

CO-INVESTIGATOR(S):
Quynh Doan

SPONSORING AGENCIES:
- Lawson Health Research Institute

PROJECT TITLE:
Anxiolysis for Laceration repair in children: An open-label multicenter adaptive trial (ALICE)

REMINDER: The current UBC Children's and Women's approval for this study expires: October 23, 2025

The UBC Children's and Women's Research Ethics Board has reviewed the above described research project, including associated documentation noted below, and finds the research project acceptable on ethical grounds for research involving human subjects and hereby grants ethics approval. Note that institutional approval is required before research can commence at C&W.

This approval applies to research ethics issues only. The approval does not obligate an institution or any of its departments to proceed with activation of the study. The Principal Investigator for the study is responsible for identifying and ensuring that resource impacts from this study on any institution are properly negotiated, and that other institutional policies are followed. The REB assumes that investigators and the coordinating office of all trials continuously review new information for findings that indicate a change should be made to the protocol, consent documents or conduct of the trial and that such changes will be brought to the attention of the REB in a timely manner.

REB FULL BOARD MEETING REVIEW DATE:	THE FOLLOWING REB MEMBERS DID NOT ATTEND THE DISCUSSION OR VOTE FOR THE ABOVE STUDY AT THIS REB MEETING REVIEW DUE TO A POTENTIAL CONFLICT OF INTEREST:
**October 23, 2024**	Srinivas Murthy

DOCUMENTS INCLUDED IN THIS APPROVAL: | **DATE DOCUMENTS APPROVED:**

Document Name	Version	Date	Date Documents Approved
**Protocol:**			
ALICE - Protocol	2.9	September 12, 2024	**December 13, 2024**
**Health Canada regulatory approval:**			
NOL284684	N/A	March 20,	

		2024	
**Consent Forms:**			
ALICE - Parent/Caregiver LOI	1.1	December 12, 2024	
ALICE - Consent Form	2.1	December 12, 2024	
ALICE - eConsent Form	2.1	December 12, 2024	
**Assent Forms:**			
ALICE - Assent Form (7-12.99 years)	2	November 13, 2024	
ALICE - eAssent Form (7-12.99)	2	November 27, 2024	
**Investigator Brochures:**			
Midazolam	1	January 3, 2023	
Dexmedetomidine	1	November 27, 2023	
Nitrous Oxide	N/A	May 15, 2017	
**Questionnaire, Questionnaire Cover Letter, Tests:**			
ALICE - PHBQ REDCap Email	2	November 27, 2024	
ALICE - PHBQ Telephone Script	N/A	October 17, 2024	
**Other Documents:**			
PERC - Peer Review 1	N/A	February 1, 2023	
PERC - Peer Review 2	N/A	February 1, 2023	
PEMRC - Peer Review	N/A	September 19, 2024	
ALICE - Screening and Eligibility Form	2.8	July 19, 2024	
Sedation Committee - Letter of Support	N/A	November 5, 2024	
ALICE - ED Enrollment Form	2.8	July 19, 2024	
ALICE - Vital Signs	2.8	July 19, 2024	
ALICE - DSMB Charter	2	November 27, 2023	
ALICE - Therapy Administration Checklist and Post-Therapy Beside Monitorring	2.8	July 19, 2024	
ALICE - Post Hospital Behavior Questionnaire	2.8	July 19, 2024	
ALICE - Outcome Assessor Data Collection Form	2.8	July 19, 2024	
ALICE - Informed Consent Confirmation Form	2.8.1	November 27, 2024	
ALICE - Post Hospital Behavior Questionnaire - REDCap Form	2.8	July 19, 2024	
Guidelines for Pediatric Sedation	C-05-12-62350	February 14, 2024	
ALICE - Contact Information Sheet	2.8.1	November 27, 2024	

CERTIFICATION:

In respect of clinical trials:

1. The membership of this Research Ethics Board complies with the membership requirements for Research Ethics Boards defined in Division 5 of the Food and Drug Regulations.

2. The Research Ethics Board carries out its functions in a manner consistent with Good Clinical Practices.
3. This Research Ethics Board has reviewed and approved the clinical trial protocol and informed consent form for the trial which is to be conducted by the qualified investigator named above at the specified clinical trial site. This approval and the views of this Research Ethics Board have been documented in writing.

The documentation included for the above-named project has been reviewed by the UBC Children's and Women's Research Ethics Board, and the research study, as presented in the documentation, was found to be acceptable on ethical grounds for research involving human subjects and was approved by the UBC Children's and Women's Research Ethics Board.

Approved by one of:

Dr. Holly Longstaff, Chair

Dr. Caron Strahlendorf, Associate Chair
