# Supplementary material for: Anxiolysis for laceration repair in children: study protocol for an open-label multicenter adaptive trial (ALICE)
Source: PLoS One. 2025 Jun 4;20(6):e0324515. doi: 10.1371/journal.pone.0324515 (PMC12136299; doi:10.1371/journal.pone.0324515)
Supplement: S9 File — (PDF) [file pone.0324515.s009.pdf]

Date: 10 February 2025

To: Dr Naveen Poonai

Project ID: 120985

Review Reference: 2025-120985-104864

Study Title: Anxiolysis for laceration repair in children: An open-label multicenter adaptive trial (ALICE)

Application Type: HSREB Amendment Form

Review Type: Delegated

Full Board Reporting Date: 25/Feb/202523/Apr/2024 13:00

Date Approval Issued: 10/Feb/2025 18:56

REB Approval Expiry Date: 17/Nov/2025

Dear Dr Naveen Poonai ,

The Western University Health Sciences Research Ethics Board (HSREB) has reviewed and approved the WREM application form for the amendment, as of the date noted above.

Documents Approved:

Document Name	Document Type	Document Date	Document Version
1.7.1.1.1 ALICE SPIRIT Protocol v3.0 07Jan2025-CL	Protocol	07/Jan/2025	3.0
1.7.1.2 ALICE SPIRIT Protocol v3.1 27Jan2025-CL	Protocol	27/Jan/2025	3.1
1.7.2.2 Main ICF v3.6 ALICE 27Jan2025-CL	Consent Form	27/Jan/2025	3.6
1.7.2.6 LOI Parent v1.5 ALICE 27Jan2025-CL	Consent Form	27/Jan/2025	1.5
1.7.2.4 Assent v2.3 ALICE 27Jan2025-CL	Assent Form	27/Jan/2025	2.3
1.7.1.2.2 CRF ALICE -07Jan2025-CL	Other Data Collection Instruments	07/Jan/2025	3.0

Documents Acknowledged:

Document Name	Document Type	Document Date	Document Version
1.0.1.1 CTA-A Cover Letter	Sponsor Correspondence	13/Jan/2025	
1.2.1.1 3011 form	Sponsor Correspondence	13/Feb/2025	
1.2.5.1.1 CTSI NTF	Sponsor Correspondence	07/Jan/2025	
1.0.1.1 CTA-N Cover Letter	Sponsor Correspondence	31/Jan/2025	
Acknowledgement of receipt of CTA-N protcol 3.1_03Feb2025	Sponsor Correspondence	03/Feb/2025	
NOL294214	NOL/NOA/ITA/CTA	24/Jan/2025	
2.3.1 Pharmacy Manual ALICE v1.2 04Dec2024-CL	Other Materials	04/Dec/2024	1.2
1.7.1.1.3 Summary of Changes v3.1_27Jan2025	Summary of Changes	27/Jan/2025	

Document Name	Document Type	Document Date	Document Version
1.7.1.1.1 ALICE SPIRIT Protocol v3.0 07Jan2025-TK	Summary of Changes	07/Jan/2025	3.0
1.7.1.1.1 ALICE SPIRIT Protocol v3.1 27Jan2025-TK	Summary of Changes	27/Jan/2025	3.1

REB members involved in the research project do not participate in the review, discussion or decision.

The Western University HSREB operates in compliance with, and is constituted in accordance with, the requirements of the TriCouncil Policy Statement: Ethical Conduct for Research Involving Humans (TCPS 2); the International Conference on Harmonisation Good Clinical Practice Consolidated Guideline (ICH GCP); Part C, Division 5 of the Food and Drug Regulations; Part 4 of the Natural Health Products Regulations; Part 3 of the Medical Devices Regulations and the provisions of the Ontario Personal Health Information Protection Act (PHIPA 2004) and its applicable regulations. The HSREB is registered with the U.S. Department of Health & Human Services under the IRB registration number IRB 00000940.

Please do not hesitate to contact us if you have any questions.

Electronically signed by:

Nicola Geoghegan-Morphet , Ethics Officer on behalf of Dr. Roberta Berard, HSREB Vice-Chair, 10/Feb/2025 18:56

Reason: I am approving this document.

Note: This correspondence includes an electronic signature (validation and approval via an online system that is compliant with all regulations).
